# Comparative Transcriptome Profiling of the Early Response to *Magnaporthe oryzae* in Durable Resistant *vs* Susceptible Rice (*Oryza sativa* L.) Genotypes

**DOI:** 10.1371/journal.pone.0051609

**Published:** 2012-12-12

**Authors:** Paolo Bagnaresi, Chiara Biselli, Luigi Orrù, Simona Urso, Laura Crispino, Pamela Abbruscato, Pietro Piffanelli, Elisabetta Lupotto, Luigi Cattivelli, Giampiero Valè

**Affiliations:** 1 Consiglio per la Ricerca e la Sperimentazione in Agricoltura-Genomics Research Centre, Fiorenzuola d’Arda, Piacenza, Italy; 2 Consiglio per la Ricerca e la Sperimentazione in Agricoltura-Rice Research Unit, Vercelli, Italy; 3 Parco Tecnologico Padano, Lodi, Italy; 4 Consiglio per la Ricerca e la Sperimentazione in Agricoltura-Department of Plant Biology and Crop Production, Roma, Italy; Ghent University, Belgium

## Abstract

Durable resistance to blast, the most significant fungal disease of rice, represents an agronomically relevant character. Gigante Vercelli (GV) and Vialone Nano (VN) are two old temperate *japonica* Italian rice cultivars with contrasting response to blast infection: GV displays durable and broad resistance while VN is highly susceptible. RNA-seq was used to dissect the early molecular processes deployed during the resistance response of GV at 24 h after blast inoculation. Differential gene expression analysis identified 1,070 and 1,484 modulated genes, of which 726 and 699 were up regulated in response to infection in GV and VN, respectively. Gene ontology (GO) enrichment analyses revealed a set of GO terms enriched in both varieties but, despite this commonality, the gene sets contributing to common GO enriched terms were dissimilar. The expression patterns of genes grouped in GV-specific enriched GO terms were examined in detail including at the transcript isoform level. GV exhibited a dramatic up-regulation of genes encoding diterpene phytoalexin biosynthetic enzymes, flavin-containing monooxygenase, class I chitinase and glycosyl hydrolase 17. The sensitivity and high dynamic range of RNA-seq allowed the identification of genes critically involved in conferring GV resistance during the early steps of defence perception-signalling. These included chitin oligosaccharides sensing factors, wall associated kinases, MAPK cascades and WRKY transcription factors. Candidate genes with expression patterns consistent with a potential role as GV-specific functional resistance (*R*) gene(s) were also identified. This first application of RNA-seq to dissect durable blast resistance supports a crucial role of the prompt induction of a battery of responses including defence-related genes as well as members of gene families involved in signalling and pathogen-related gene expression regulation.

## Introduction

World-wide rice production is severely affected by rice blast, a major fungal disease caused by *Magnaporthe oryzae*, which can lead to yield losses of up to 50–70% [1], [2]. The blast fungus mechanically breaches the leaf epidermis using an appressorium, a dome shaped cell that generates enormous turgor pressure [3], [4]. The appressorium produces a specialized hypha, a penetration peg, which pierces the leaf surface. Upon reaching the epidermal cell lumen, the penetration peg expands to form a narrow filamentous primary hypha. In compatible interactions, primary hyphae differentiate into thicker, bulbous invasive hyphae that fill the initially invaded cells and then move into neighbouring cells [5], [6]. *M. oryzae* has been described as a hemibiotrophic pathogen [4]. The initial plant cell invasion is defined as biotrophic, because invaded cells appear healthy and retain the ability to plasmolyze [7]. A necrotrophic phase follows, during which the pathogen induces host cell death. In compatible interactions the fungus penetrates the host cells at 24 h post inoculation (hpi), then invasive hyphae grow in the first invaded cell for 8 to 12 hours, often filling it up. A time-dependent switch occurs during this period because invasive hyphae spread into neighbouring cells between 32 and 36 hpi no matter how quickly, or how completely, the first cell has been filled with fungus [6]. Compatible rice cells containing invasive hyphae plasmolyze at 27 hpi, suggesting that the cell’s plasma membrane is intact and functional at this time, while they fail to plasmolyze after 32 hpi, indicating that the plasma membrane is no longer active and the cell is not viable when the fungus moves to the neighbouring cells [6]. During a major resistance gene-mediated incompatible interaction, most invaded host cells fail to plasmolyze within 27 hpi suggesting that at this time point resistance gene-dependent defence reactions have already damaged membrane integrity [6], [7]. Molecular analyses before 27 hpi in a resistant genotype should therefore highlight defence pathways activated during the resistance response.

Breeding for durable blast resistance is a difficult task due to both the high degree of pathogenic variability of *M. oryzae* [8] and the large number of fungal races encountered in the field population. Currently, more than 70 blast disease resistance (R) genes have been identified [9], [10], but evolution of the pathogen necessitates continuous screening efforts to uncover new broad spectrum and durable sources of resistance. Several blast R genes, all located in the same region of rice chromosome 6, confer broad-spectrum resistance to different sets of blast strains, e.g. *Pi9*, *Pi2* and *Piz-t* [11]–[15]. Partial resistance conferred by QTLs is generally more durable and non-race specific [16], and up to now several blast resistance QTLs have been mapped (reviewed in [10]), while the genes underlying two of them, *pi21* and *Pb1*, have also been cloned [17], [18].

A dissection of the molecular processes deployed by R genes during blast infection have been already investigated using microarrays in several experimental systems: e.g. rice carrying the R gene *Pi33* infected with *M. oryzae* strain carrying the avirulence gene *ACE1* [19], resistant *vs.* susceptible NILs differing for the introgression of the *Pi-k* gene [20], and genotypes carrying partial resistance genes [21]. A collection of ESTs from resistant, partially resistant and susceptible infected rice leaves has also been created [22]. These studies allowed the identification of the main biological processes and functional categories of defence-related genes responsive to rice blast. Nevertheless, to the best of our knowledge, the whole transcriptome sequencing (RNA-Seq) approach has never been used to compare defence responses in resistant *vs.* susceptible rice-blast interactions. RNA-Seq is increasingly used for gene expression profiling in plants [23]–[25] as it provides significant advantages over traditional microarray analysis: e.g. accurate quantification of gene expression with low background, high sensitivity and the capability of detecting differential expression over a large dynamic range, high reproducibility for both technical and biological replicates as well as the possibility of detecting novel splicing isoforms and boundaries of un-translated regions at single nucleotide resolution [26], [27].

Despite being poorly characterized, Gigante Vercelli (GV), an old Italian variety released in 1946 (*japonica* group), exhibits a considerable durable and broad spectrum blast resistance that has lasted for more than 60 years. Upon infection, GV typically shows many pin-point lesions without further progress of the infection process [28], [29]; current genetic investigations suggest that major resistance gene(s) govern GV response to blast (unpublished data).

In this work, RNA-Seq was used to identify early post inoculation transcriptional changes distinct to GV as compared to Vialone Nano (VN), a fully blast susceptible cultivar. GV-specific response was characterized by a prompt induction and fine regulation of a battery of processes including up-regulation of diterpene phytoalexin biosynthetic (DPB) genes, perception and signalling through wall associated kinases (*WAKs*), chitin sensors, *WRKY* and *MAP*(*KKK*) genes families, activation of defence genes such as chitinase, glycosyl hydrolase and a regulated expression of resistance gene analogs.

## Results and Discussion

### Identification of Blast Infection-related Differentially Expressed Genes (DEG)

RNA isolated from leaves of Gigante Vercelli (GV) and Vialone Nano (VN), 24 h after inoculation (hai) with the blast fungus *Magnaporthe oryzae* or after mock inoculation, was subjected to paired-end whole transcriptome sequencing. Three biological replicates were perfomed for each rice genotype (GV *vs* VN) and condition (mock *vs* blast-inoculated). The raw reads obtained from the Illumina Genome Analyzer IIx were filtered (Illumina passed-filter call) and further checked for sequence contaminants with the fastQC application About 40 million pairs of filtered 37 base reads obtained for each biological replicate (Table 1), were mapped with Bowtie/TopHat to the rice genome sequence (*O. sativa* Japonica Group cultivar Nipponbare; ENSEMBL release 6.12). Assuming a transcriptome size of about 100 Mb (1,444 bp median length × 68,682 transcripts; ENSEMBL cDNA release 6.12), we attained a mean coverage of 27× per biological replicate.,In agreement with recent rice RNA-seq experiments [30], a relevant fraction of reads (from 8 to 23% of total mapped reads; Table 1) corresponded to intergenic regions, suggesting the presence of as yet uncharacterized transcriptionally active regions. Upon deduplication of PCR duplicates, read counts were obtained from SAM alignment files with HTSeq software. Differentially expressed genes (DEGs) were called with DESeq. An RPKM (Reads per Kilobase per Million) cutoff value of 0.1 was set to declare a locus expressed [31] resulting in 30,890 and 28,109 loci passing the expression cutoff for GV and VN, respectively. High Spearman correlation coefficients of the expression values (>0,95) were observed when genotypes and treatments were compared, indicating a good level of reproducibility among biological replicates (Figure S1).

**Table 1:** Numbers of aligned and mapped reads.

Samples	Total filtered Pair-end reads	Total mapped reads	Reads mapping in intergenic regions	Aligned bases	Uniquely mapping reads
**GV** a **mock 1**	2×41,647,574	78,544,360	18,146,784	2,906,141,320	41,075,176
**GV mock 2**	2×44,165,006	83,144,389	13,433,235	3,076,342,393	43,449,446
**GV mock 3**	2×42,642,301	80,326,551	14,438,901	2,972,082,387	42,048,286
**GV blast 1**	2×37,886,777	71,008,350	11,889,351	2,627,308,950	37,132,384
**GV blast 2**	2×44,572,662	82,648,615	14,875,748	3,057,998,755	43,779,698
**GV blast 3**	2×34,171,043	63,636,891	10,899,064	2,354,564,967	33,587,892
**VN** a **mock 1**	2×43,315,664	81,797,448	12,594,315	3,026,505,576	42,363,534
**VN mock 2**	2×42,782,371	80,418,573	8,181,024	2,975,487,201	41,718,338
**VN mock 3**	2×32,326,921	60,941,220	9,072,029	2,254,825,140	31,553,233
**VN blast 1**	2×41,169,424	76,528,664	7,091,526	2,831,560,568	39,854,171
**VN blast 2**	2×33,448,669	61,907,658	5,993,239	2,290,583,346	32,258,965
**VN blast 3**	2×31,139,332	57,774,685	4,513,106	2,137,663,345	30,091,261

a GV =  Gigante Vercelli; VN =  Vialone Nano.

**Figure 1:**
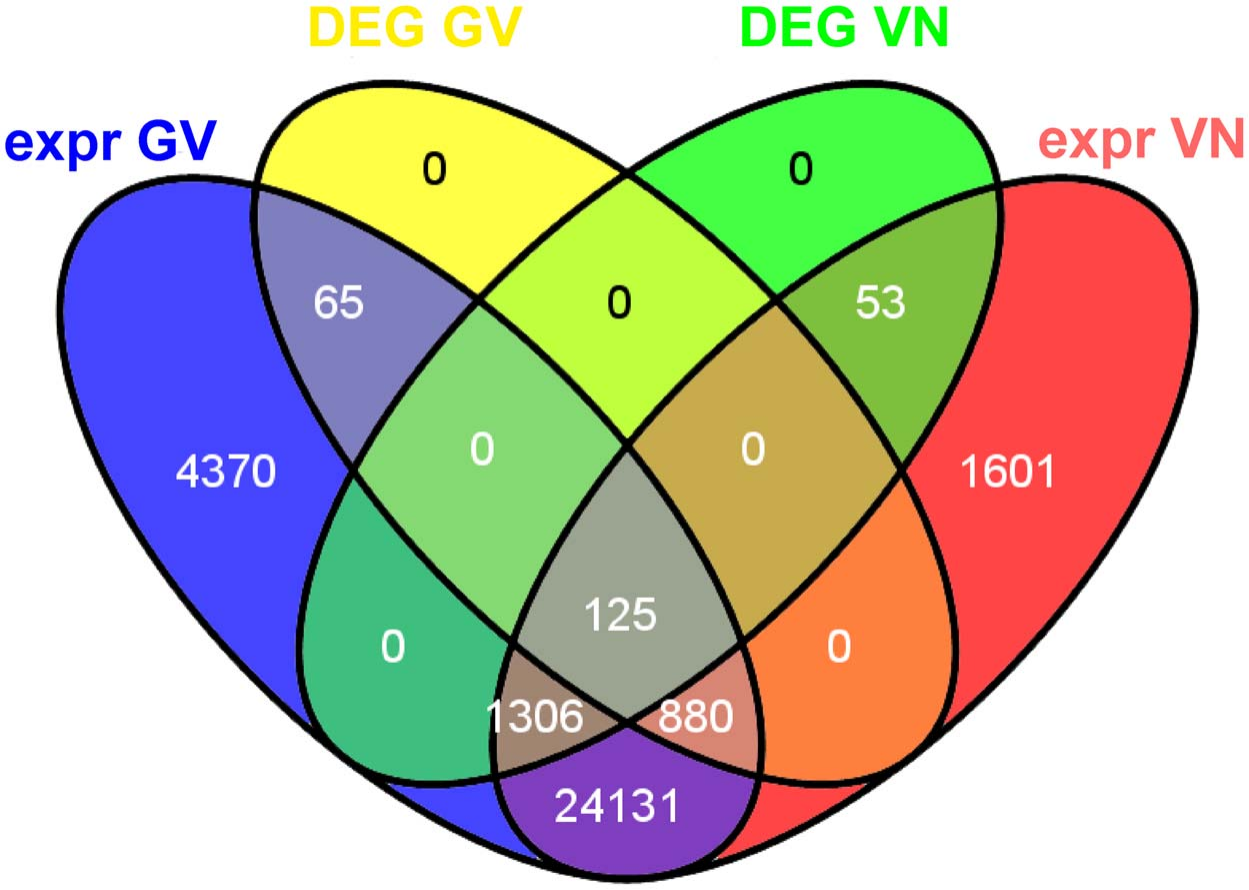
Venn diagrams of total and blast-modulated genes. Venn diagrams illustrating the relationships between total expressed (expr) genes and DEGs after blast inoculation in the two rice genotypes Gigante Vercelli (GV) and Vialone Nano (VN).

**Figure 2:**
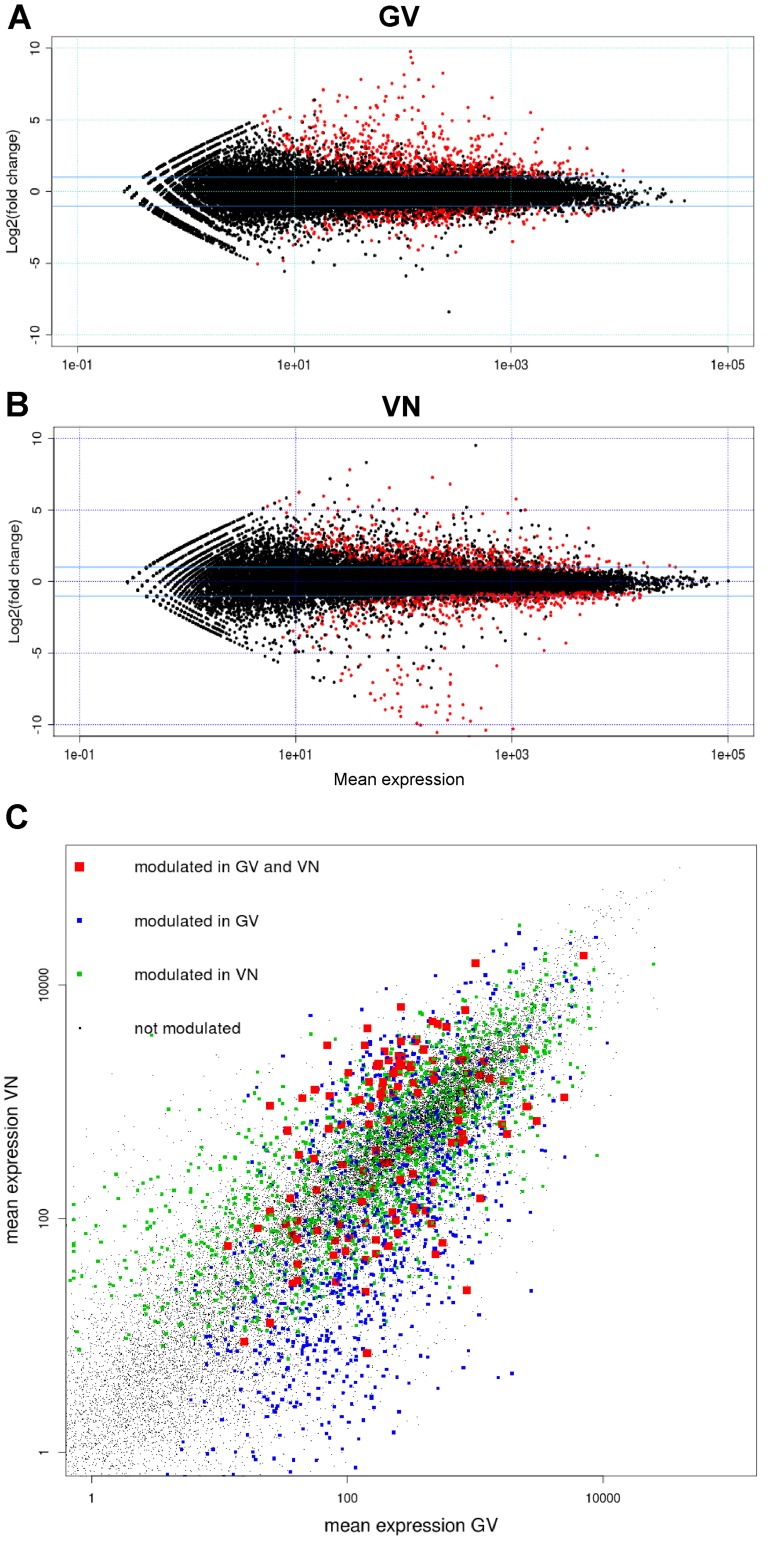
Global overview of Gigante Vercelli (GV) and Vialone Nano (VN) transcriptional changes. Mean expression *versus* log fold change plots (MA-plots) were computed for GV (A) and VN (B); normalised expression mean values are plotted versus log2 fold changes and called DEGs (FDR <0.05) are plotted in red. (C) Mean expression values (averages of mock and infected) were plotted on x and y axis for GV and VN, respectively. Genes called as DEG in both varieties, in GV only, in VN only or in neither genotype are plotted in red, blue, green and black, respectively.

For the calling of DEGs, the False Discovery Rate (FDR) threshold was set to 0.05 and a combination of fitted versus per-gene dispersion values (Figure S2) was implemented as suggested by the default DESeq settings. As shown in the Venn diagram in Figure 1, 1,070 DEGs were identified in GV (of which 65 were GV-specific expressed loci, 125 were also called as DEGs in VN and 880 were genes called as DEGs in GV only but expressed also in VN), while 1,484 DEGs were identified in VN (consisting of 53 VN-specific expressed loci, 125 common DEGs and 1306 called as DEGs in VN only but as expressed loci also in GV). It is notable that the defence responses of the two genotypes were largely different, with only 125 DEGs in common, and that the number of DEGs was higher in the susceptible (VN) than in the resistant genotype (GV). The latter observation may be attributed to the successful establishment of infection in the susceptible variety resulting in complex adaptive mechanisms to the hemibiotrophic lifestyle of *M. oryzae* ([32], and references therein). However, mean expression *versus* log fold change plots (MA-plots) (Figure 2A, B) illustrated that among the DEGs, the proportion of up-regulated genes was higher in GV (726 or 67.8%) than in VN (699 or 47.1%). Indeed, a global view of the genes monitored during the experiments indicated that a substantial down-regulation of genes occurred in VN, contributing to the higher number of modulated genes in the blast susceptible genotype (Figure 2C). Down-regulated genes, in both GV and VN, were principally involved in photosynthesis-related processes and metabolism of photosynthesis-associated compounds. In down-regulated VN genes, a particular emphasis on chloroplast-related and carotenoid biosynthetic genes (GO:0016117) was observed. Photosynthetic and carotenoid genes were previously shown to be down-regulated during *M. grisea* infection in both compatible and incompatible interactions ([19] and references therein; [20]) and the reater extent of down-regulation of these GO groups in VN with respect to to GV is likely related to the fast infection progression in the susceptible genotype. Expression values for DEGs and all the transcribed genes detected in the experiment are reported in Table S1.

### GO Enrichment Analysis

GO enrichment analysis in RNA-Seq experiments poses specific challenges as the status of modulated genes (i.e. DEG classification) is related to the read counts and thus biases in favour of longer and highly expressed genes are expected [33]. The length-related bias, in particular, cannot be neglected as for microarray-based expression studies where probes are of similar or identical length within a given platform. The goseq R package [33] has been conceived to address these RNA-Seq specific biases and, to this end, requires the input of gene lengths based on the median length of transcripts associated with each locus.

**Figure 3:**
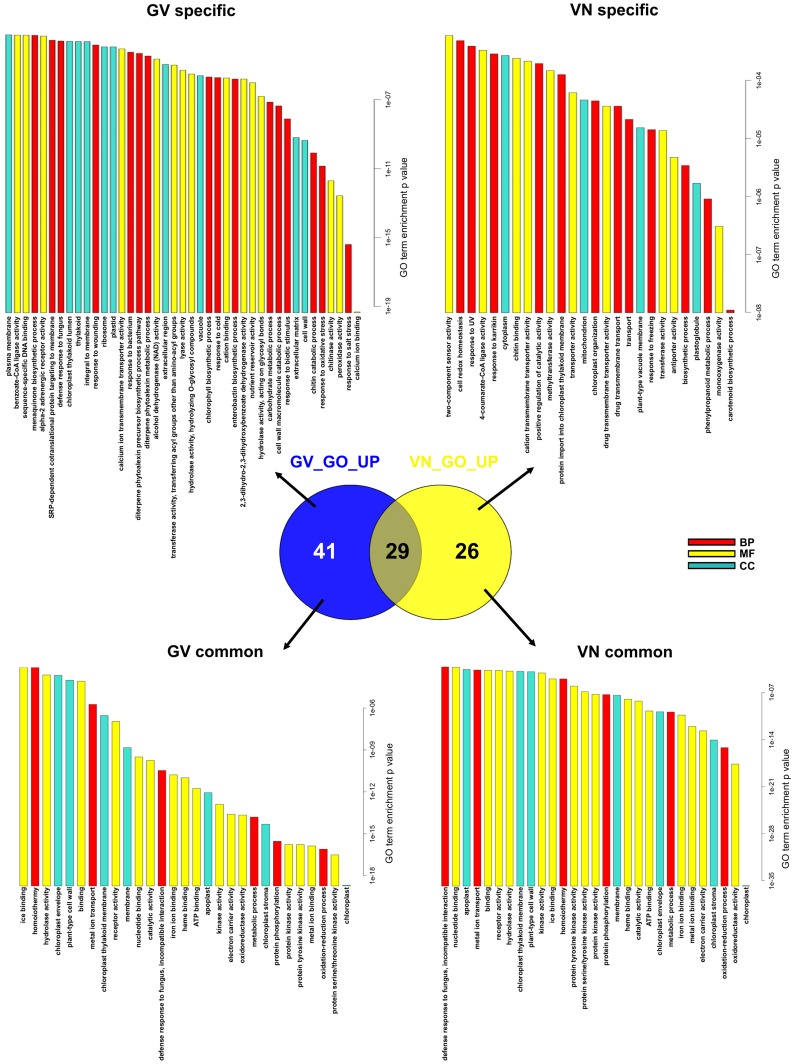
GO enrichment in Gigante Vercelli (GV) and Vialone Nano (VN). Common, GV-specific and VN-specific enriched GO terms as estimated by goseq at an FDR cutoff of 0.05. Enrichment p values are shown on X axis. Two bar plots are shown for the common enriched GO to detail the distinct p values for the varieties. BP (red): Biological Process; MF (yellow): Molecular Function; CC (turquoise): Cell Compartment.

For both varieties, the DEGs estimated by DESeq package were used as a test set for goseq input. Goseq output (threshold FDR < = 0.05) yielded 70 and 55 enriched GO terms for GV and VN, respectively (Figure 3). Common (29 GO), GV-specific (41 GO) and VN-specific (26 GO) enriched terms were identified, highlighting a substantial higher number of terms associated to the resistant response in comparison to the susceptible one. Several of the 29 enriched terms shared by the two rice genotypes were consistent with responses to fungal infection; these included “defence response to fungus, incompatible interaction” (GO:0009817), “apoplast” (GO:0048046), “cell wall” (GO:0005618, connected to defence-related secreted proteins and cell wall reinforcement) and oxido-reductive reactions (GO:0016491; GO:0055114; GO:0009055), typical terms of defence-related oxidative bursts and reactive oxygen species (ROS) generation/detoxification. However, despite these similarities, careful consideration of the individual genes contributing to the common enriched GO terms revealed substantial diversity between genotypes. For example, 13 and 7 genes in the group “defence response to fungus, incompatible interaction” (51 genes in GO release MSU6.12) were modulated in GV and VN, respectively, but only 3 of them, two putative thaumatins and WIP3 (Wound-Induced Protein precursor) were shared between the two genotypes (Table 2). Apart from this GO group, gene-level dissection of further shared GO terms enriched in both varieties showed that the gene pool contributing to the enrichment of the common categories was heterogeneous between GV and VN. This observation reflects the distinct responses of the two varieties to infection.

**Table 2:** Differentially Expressed Genes in the common enriched GO term “defence response to fungus, incompatible interaction” (GO:0009817) in Gigante Vercelli and Vialone Nano.

		Gigante Vercelli					Vialone Nano				
id	MSU `6.12 description	Mock inoculated^a^	Blast inoculated^a^	Fold change	FDR	Listed among DEGs	Mock inoculated^a^	Blast inoculation	Fold change	FDR	Listed among DEGs
LOC_Os01g51570	glycosyl hydrolases family 17, putative,expressed	2927.53	8100.54	2.77	4.27E−004	YES	336.86	2883.61	8.56	0.16	NO
LOC_Os01g71340	glycosyl hydrolases family 17. putative,expressed	5717.52	15734.46	2.75	3.33E−004	YES	621.97	5817.86	9.35	0.25	NO
LOC_Os01g71350	glycosyl hydrolases family 17, putative,expressed	1369	4236.71	3.09	6.85E−005	YES	120.22	1100.76	9.16	0.24	NO
LOC_Os01g71474	glycosyl hydrolases family 17, putative, expressed	15.29	122.02	7.98	4.83E−009	YES	1.53	1.37	0.89	1	NO
LOC_Os01g71670	glycosyl hydrolases family 17, putative,expressed	240.41	835.55	3.48	0.02	YES	1323.68	5325.68	4.02	0.09	NO
LOC_Os01g71830	glycosyl hydrolases family 17, putative,expressed	23.4	436.62	18.66	0.01	YES	0.42	2.54	6.12	0.81	NO
LOC_Os03g15050	phosphoenolpyruvate carboxykinase,putative, expressed	3630.94	7788.02	2.14	0.04	YES	5197.28	7874.69	1.52	0.11	NO
LOC_Os03g45960	thaumatin, putative, expressed	70.57	867.08	12.29	6.81E−006	YES	51.75	360.83	6.97	0.04	YES
LOC_Os03g46070	thaumatin, putative, expressed	280.98	3030.45	10.79	6.37E−016	YES	171.92	1293.07	7.52	0.26	NO
LOC_Os03g46440	BTBA4 - Bric-a-Brac,Tramtrack, BroadComplex BTB domain with Ankyrinrepeat region, expressed	470.44	1453.45	3.09	2.98E−005	YES	515.18	812.6	1.58	0.96	NO
LOC_Os05g31140	glycosyl hydrolases family 17,putative, expressed	6726.72	4776.57	0.71	0.89	NO	2195.96	7501.05	3.42	3.31E−008	YES
LOC_Os06g14510	glucose-6-phosphate isomerase,putative, expressed	342.82	267.34	0.78	0.91	NO	1850.33	906.93	0.49	2.19E−003	YES
LOC_Os08g41730	peptidase, T1 family, putative, expressed	89.03	109.77	1.23	0.98	NO	185.57	489.98	2.64	0.01	YES
LOC_Os11g37950	WIP3 - Wound-induced protein precursor, expressed	54.82	463.3	8.45	5.97E−013	YES	28.07	404.86	14.42	0.01	YES
LOC_Os11g37960	WIP4 - Wound-induced proteinprecursor, expressed	185.27	973.62	5.26	7.97E−011	YES	74.53	548.98	7.37	0.11	NO
LOC_Os11g37970	WIP5 - Wound-induced proteinprecursor, expressed	166.97	303.3	1.82	0.62	NO	489.97	2095.05	4.28	0.02	YES
LOC_Os12g43450	thaumatin family domain containingprotein, expressed	92.21	400.73	4.35	0.01	YES	318.5	2374.89	7.46	0.04	YES

a Expression values are reported as DESeq-normalized read counts.

Conversely, several GO terms were genotype-specific. The term “defence response to fungus” (GO:0050832) was enriched solely in GV, since genes involved in the general fungal defence responses, including activation of flavin-containing mono-oxygenase and chitinases, were found in GV but not in VN (see Table S1 and Table 6). Additionally, only GV exhibited enrichment in further terms, including “diterpene phytoalexin metabolic process (GO:0051501), “diterpene phytoalexin precursor biosynthetic process pathway” (GO:0051504), “calcium ion binding” (GO:0005509), “peroxidase activity” (GO:0004601), “chitinase activity” (GO:0004568), “response to oxidative stress”, (GO:0006979) and “chitin catabolic process” (GO:0006032). Furthermore, when only up-regulated DEGs were considered for both GV and VN, a further GV-specific enriched GO term was revealed, namely “sequence-specific DNA binding transcription factor activity” (GO:0003700). The above GO groups, being GV-specific, are promising candidates for revealing genes underpinning GV resistance and are examined in more detail in the following sections.

### Diterpene Phytoalexin Genes Group

In rice, 15 phytoalexins (PA) have been characterized [34] and much effort has been dedicated to the characterization of the diterpene phytoalexins in particular. Experimental evidence points to leaf accumulation of the diterpene phytoalexins phytocassanes, momilactones and oryzalexins as critical responses in counteracting *M. oryzae* infection [35], [36], [37]. Upon blast infection in rice, these compounds accumulate specifically at the edges of necrotic lesions preventing fungal spore germination and germ tube growth [37], [38]. Diterpene phytoalexin biosynthetic genes (DPB genes) have been found up-regulated in cultured rice cells triggered with elicitors (chitin oligosaccharides). The genes *OsCPS2* and *OsKSL7* are involved in the biosynthesis of phytocassanes, *OsCPS4* and *OsKSL4* in the biosynthesis of momilactones, and *OsKSL10* and *OsKLS8* in oryzalexin biosynthesis. Interestingly, several of these DPB genes are clustered in two regions on chromosome 4 (momilactones) and chromosome 2 (phytocassanes).

**Figure 4:**
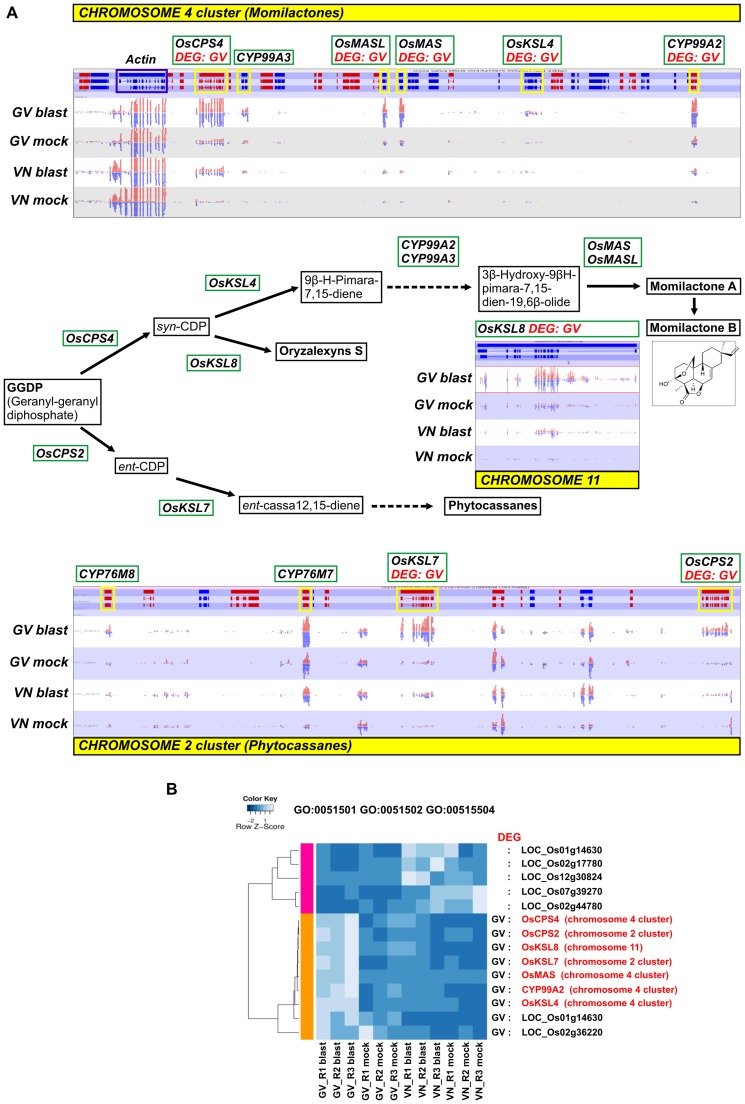
Modulation of DPB genes in Gigante Vercelli (GV) and Vialone Nano (VN). **A:** Diterpene phytoalexin gene clusters on the rice chromosome 4 (upper panel) and 2 (lower panel). Read mapping patterns of the *OsKSL8* gene on rice chromosome 11 are also reported. Genes called as DEGs are indicated below gene symbols and genes boxed in yellow in the panels representing gene, mRNA and coding sequence structures (respectively from the upper to the lower bars) are known/candidate diterpene phytoalexin biosynthetic genes (*DPB* genes). Reads from the 3 biological replicates for each genotype and treatment were merged and visualized with Seqmonk software. Red and blue segments above and below the midlines represent reads mapping to forward and reverse strands, respectively. The actin gene flanking the chromosome 4 cluster (boxed in blue) is shown as an internal control. In the middle panel, genes involved in the diterpene phytoalexin biosynthetic pathways are boxed in green and compounds in black. **B**: Hierarchical clustering of DPB genes based on their expression values. The reported DPB genes are those listed (MSU release 6.12) to GO terms GO:0051501 (diterpene phytoalexin metabolic process), GO:0051502 (diterpene phytoalexin biosynthesis) and GO:0051504 (diterpene phytoalexin precursor biosynthetic process pathway) in the different GV and VN treatments (blast or mock inoculated) and biological replicates (R1, R2, R3). The colour scale indicates the expression value (light blue indicate higher expression value, darker blue indicates lower gene expression values). The names of the genes in common with panel A are highlighted in red. Genes called as DEGs and their chromosome cluster location are indicated on the right border of the heatmap. Colored bars on the left of the heatmap mark distinct major branches in the clustering tree grouping genes with similar expression pattern.

At 24 h hai a strong induction of the key biosynthetic genes localized in the two chromosome clusters (Figure 4A) was observed only in GV. OsMASL, CYP76M8 and CYP76M7, which are DPB genes recently included in chromosome 2 and 4 clusters (Okada, 2011) but not yet included in diterpene phytoalexin GO groups, were also recovered as DEGs. Furthermore, the DPB gene *OsKSL8* (LOC_Os11g28530; oryzalexin S synthesis) on chromosome 11 showed a basal level of gene expression (mock inoculated conditions) consistently higher in GV than in VN and, after blast infection, it was significantly up-regulated in the resistant genotype (4.5-fold induction) but not in the susceptible one (despite some induction trend observed in VN).

The ensemble of DPB genes is defined by GO terms GO:0051501 (diterpene phytoalexin metabolic process), GO:0051502 (diterpene phytoalexin biosynthesis) and GO:0051504 (diterpene phytoalexin precursor biosynthetic process pathway). A heatmap of this gene set in Figure 4B highlights the up-regulation of crucial genes belonging to the diterpene phytoalexin biosynthetic pathway in the resistant genotype GV. Expression values for differentially expressed DPB genes are reported in Table S2. A similar DPB gene induction, particularly of the clusters on chromosome 4 (momilactones) and chromosome 2 (phytocassanes), was recently observed in the comparison between two Near Isogenic Lines (NILs) differing for the *pi-i* blast resistance gene. In this work, diterpene gene clusters induction occurred at 2 dpi in the resistant NIL, while in the susceptible NIL it was only evident at 4 dpi [39]. After 4 dpi, the activation of the DPB genes was similar in both susceptible and resistant NILs, indicating that the timing of rice diterpene phytoalexins accumulation might be critical to the containment of the effect of blast infection and ultimately to determining differences between resistant and susceptible phenotypes [39].

In the GV-VN experimental system, most of the genes encoding enzymes of the momilactone biosynthetic pathway and the two known genes encoding enzymes of the phytocassanases pathway were significantly up-regulated only in the blast resistant genotype within 24 hpi. This early gene induction, even anticipated in comparison to what has been reported by Hasegawa et al. (2010) [39] (in which increased DPB gene expression in blast resistant plants was observed from 48 hpi onwards), support a crucial role of DPB genes in GV blast resistance. In addition, the up-regulation of the oryzalexin S biosynthetic gene (*OsKSL8*) in GV was at least two times higher than reported previously for a genotype bearing the *Pi−i* resistance gene at the same time point of inoculation [39], suggesting that the R gene(s) carried by GV may lead to the accumulation of a wider spectrum of diterpene phytoalexins (oryzalexin S, phytocassanes and momilactones).

It has previously been shown that, in rice cell culture, the bZIP transcription factor *OsTGAP1* (LOC_Os04g54474), participates in diterpene phytoalexin gene cluster transcriptional induction showing an about 3-fold induction after elicitation with chitin oligosaccharides [40]. In our dataset, *OsTGAP1* was expressed in both GV and VN, but not listed among DEGs. Nevertheless, beside *OsTGAP1*, additional regulatory mechanisms are required for DPB gene induction since the constitutive over-expression of *OsTGAP1* alone led to very low induction of DPB genes, and relevant expression was only attained by simultaneous elicitation of cultured cells with chitin oligosaccharide [40]. In this context, the WRKY transcription factors, previously indicated as involved in controlling phytoalexin genes expression [41], [42], are promising candidates for co-regulators of DPB genes and may further contribute to GV-specific defence mechanisms.

### Differential Modulation of WRKY Gene Family Members

**Figure 5:**
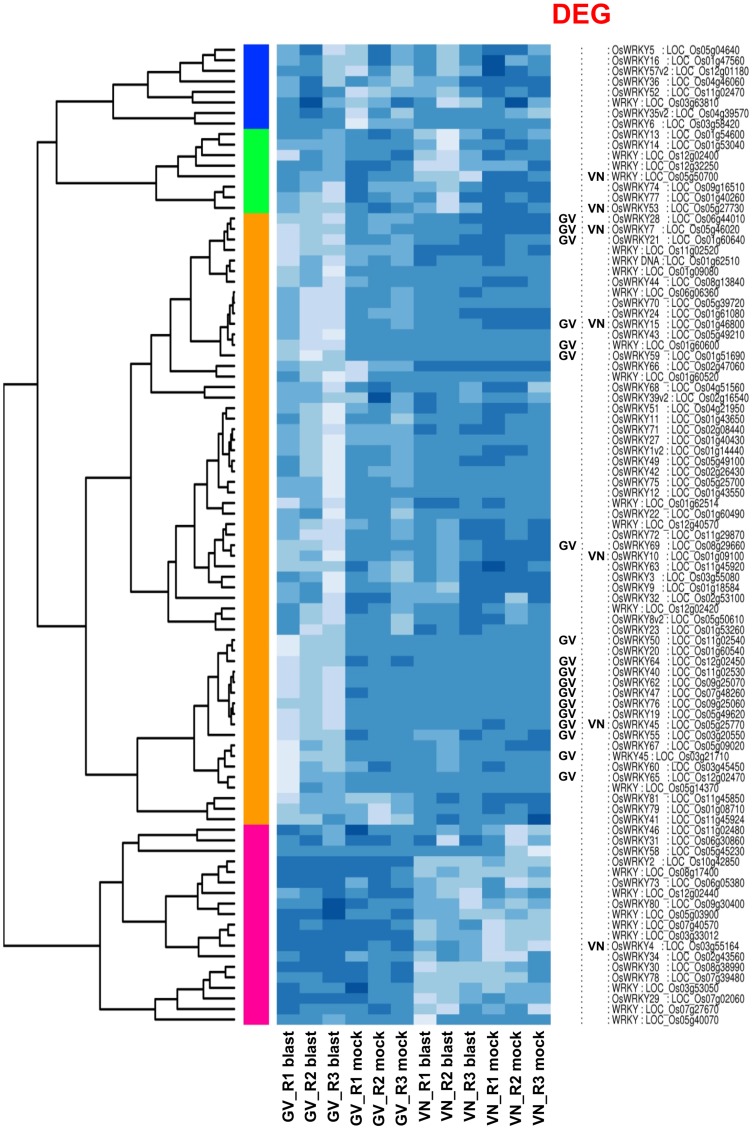
Clustering and heatmap of *WRKY* gene expression. Gigante Vercelli (GV) and Vialone Nano (VN) *WRKY* gene expression as affected by blast infection in the different treatments (blast or mock inoculated) and biological replicates (R1, R2, R3). Genes called as DEGs are indicated on the right. Colored bars on the left of the heatmap mark distinct major branches in the clustering tree grouping genes with similar expression pattern. The colour scale indicates the expression value (light blue indicate higher expression value, darker blue indicates lower gene expression values). The heat map was generated with custom scripts based on heatmap.2 function as available in the ‘gplots’ Bioconductor package.

The WRKY transcription factors superfamily contains more than 100 members in rice [43] and the regulation of defence responses is among the various processes regulated by this transcription factor class [44]. Interestingly, GV underwent a massive induction of WRKY genes upon infection and the transcription of 18 differentially expressed *WRKY* genes was induced with fold changes ranging from 2 to 870 in response to blast infection. Conversely, VN exhibited minor trends of *WRKY* genes induction, with only 7 *WRKYs* among DEGs with fold change values ranging from 2 to 3, with one *WRKY* (*WRKY4*) transcriptionally down-regulated (Table 3). Hierarchical clustering of *WRKY* genes (Figure 5) gives an overview of the extent of *WRKY* induction in GV and highlights a major cluster (marked in orange in Figure 5) of *WRKY* genes strongly up-regulated in GV but weakly/not up-regulated in VN. Additionally, only three significantly induced *WRKY* genes were in common between GV and VN, in agreement with the large differences in the defence gene induction observed in the two genotypes. A full list of the *WRKY* (as determined by INTERPRO id: IPR003657; “DNA-binding WRKY”) expression values detected in both genotypes is reported in Table S3.

**Table 3:** Differentially expressed *WRKY* genes in Gigante Vercelli and Vialone Nano.

Gigante Vercelli					
id	MSU 6.12 description	Mock inoculated^a^	Blast inoculated^a^	Fold change	FDR
LOC_Os11g02540	*OsWRKY50* - Superfamily of TFs having WRKY andzinc finger domains, expressed	0.27	233.70	872.05	2.34E−011
LOC_Os12g02470	*OsWRKY65* - Superfamily of TFs having WRKY andzinc finger domains, expressed	0.49	51.46	105.06	0.030
LOC_Os09g25070	*OsWRKY62* - Superfamily of TFs having WRKY andzinc finger domains, expressed	35.40	1206.28	34.07	2.86E−018
LOC_Os03g21710	*OsWRKY45* transcription factor, putative, expressed(WRKY79, according CGSNL nomenclature)	10.88	294.70	27.09	4.76E−008
LOC_Os09g25060	*OsWRKY76* - Superfamily of TFs having WRKY andzinc finger domains, expressed	80.63	1257.31	15.59	1.21E−012
LOC_Os05g49620	*OsWRKY19* - Superfamily of TFs having WRKY and zincfinger domains, expressed	20.64	285.22	13.82	3.90E−016
LOC_Os11g02530	*OsWRKY40* - Superfamily of TFs having WRKY and zincfinger domains, expressed	30.16	406.26	13.47	2.90E−019
LOC_Os05g25770	*OsWRKY45* - Superfamily of TFs having WRKY and zincfinger domains, expressed	250.41	3273.84	13.07	7.95E−016
LOC_Os07g48260	*OsWRKY47* - Superfamily of TFs having WRKY and zincfinger domains, expressed	85.19	787.49	9.24	4.29E−016
LOC_Os12g02450	*OsWRKY64* - Superfamily of TFs having WRKY and zincfinger domains	11.43	95.39	8.35	1.21E−007
LOC_Os01g60600	WRKY DNA-binding domain containing protein,expressed (WRKY108, according CGSNL nomenclature)	58.65	447.79	7.64	1.14E−005
LOC_Os01g51690	*OsWRKY59* - Superfamily of TFs having WRKYand zinc finger domains, expressed (WRKY26,according CGSNL nomenclature)	13.08	90.19	6.89	0.010
LOC_Os01g60640	*OsWRKY21* - Superfamily of TFs having WRKY andzinc finger domains, expressed	58.66	196.46	3.35	2.11E−004
LOC_Os05g46020	*OsWRKY7* - Superfamily of TFs having WRKY andzinc finger domains, expressed	592.03	1587.33	2.68	2.44E−003
LOC_Os06g44010	*OsWRKY28* - Superfamily of TFs having WRKY andzinc finger domains, expressed	316.64	836.89	2.64	3.03E−003
LOC_Os03g20550	*OsWRKY55* - Superfamily of TFs having WRKY andzinc finger domains, expressed	62.90	157.28	2.5	0.030
LOC_Os08g29660	*OsWRKY69* - Superfamily of TFs having WRKY andzinc finger domains, expressed	138.01	329.11	2.39	0.020
LOC_Os01g46800	*OsWRKY15* - Superfamily of TFs having WRKY andzinc finger domains, expressed	261.45	555.41	2.12	0.040
**Vialone Nano**					
**id**	**MSU 6.12 description**	**Mock inoculated** a	**Blast inoculated** a	**Fold Change**	**FDR**
LOC_Os05g25770	*OsWRKY45* - Superfamily of TFs having WRKY andzinc finger domains, expressed	248.42	818.37	3.29	0.04
LOC_Os05g50700	WRKY DNA-binding domain containing protein,expressed (WRKY111, according CGSNL nomenclature)	18.88	62.13	3.29	0.02
LOC_Os01g09100	*OsWRKY10* - Superfamily of TFs having WRKY andzinc finger domains, expressed	15.11	47.82	3.16	0.04
LOC_Os01g46800	*OsWRKY15* - Superfamily of TFs having WRKY andzinc finger domains, expressed	61.35	173.04	2.82	0.04
LOC_Os05g46020	*OsWRKY7* - Superfamily of TFs having WRKY andzinc finger domains, expressed	89.85	207.62	2.31	0.02
LOC_Os05g27730	*OsWRKY53* - Superfamily of TFs having WRKY andzinc finger domains, expressed	2452.43	5026.15	2.05	4.47E−003
LOC_Os03g55164	*OsWRKY4* - Superfamily of TFs having WRKY andzinc finger domains	1263.96	599.07	0.47	0.01

a Expression values are reported as DESeq-normalized read counts.

Previous microarray analyses showed induction of several *WRKY* genes as induced by *M. oryzae* and/or chitin oligosaccharide elicitors (e.g. *OsWRKY45*, *OsWRKY53*, *OsWRKY62, OsWRKY55* and *OsWRKY71*) and their over-expression was shown to confer enhanced resistance to blast infection [45]–[48]. In our experiments, these genes exhibited either a strong induction in GV and no or moderate induction in VN (as observed for *WRKY62* and *WRKY45*), or about 2-fold induction in GV and almost unchanged expression in VN (for *WRKY71* and *WRKY55*) (Table 3). *OsWRKY45* (LOC_Os05g25770) was previously shown to be strongly induced in response to blast infection, salicylic acid and benzothiazole (BTH) treatments and its over-expression strongly enhanced rice resistance to *M. grisea* and *Xanthomonas oryzae* pv. *oryzae*, while *WRKY45* gene knockdown strongly impaired BTH-induced resistance [49]. In GV, two *WRKY45* genes were identified as DEGs: LOC_Os05g25770 was 13-fold induced upon inoculation in GV (with a strong basal level of expression) and showing only 3.2 fold-induction in VN, while LOC_Os03g21710 (WRKY79 in CGSNL nomenclature; Table 3) was 27-fold induced in GV and not recovered as a DEG in VN. The constitutive over-expression of *OsWRKY45* (LOC_Os05g25770) is associated with hydrogen peroxide accumulation [45]; an enhanced accumulation of ROS would be consistent with the observed GO terms “peroxidase activity” (GO:0004601) and “response to oxidative stress” (GO:0006979) enriched in GV but not VN. The up-regulation of *WRKY45* suggests a possible defence-related upsurge of ROS and the consequent need of detoxification mechanism activation. *OsWRKY62*, known to determine suppression of some defence response (e.g. those induced during the *AVRXa21*-*Xa21* interaction, [50], was also strongly induced (34-fold) in GV only (Table 3), suggesting a fine tuning regulation of defence responses.

The involvement of *WRKY* genes in controlling phytoalexin genes expression is well established [41], [42], and is supported by the presence of functional W-boxes, the binding sites of *WRKY* proteins, in the promoters of several DPB genes of the gene clusters on chromosomes 2, 4 and 11 [51]. The strong induction of *WRKY* genes in GV may therefore contribute to both the magnitude and promptness of DPB responses which is lacking in VN.

*WRKY* genes are also known to induce several pathogenesis-related genes [47], [52] and this may account for the enrichment of the GO term “defence response to fungus” (GO:0050832) in GV. This GO term underlies the onset of more general fungal defence responses (e.g. activation flavin-containing mono-oxygenases, chitinases and glucanases) which was enriched in GV and not in VN.

**Figure 6:**
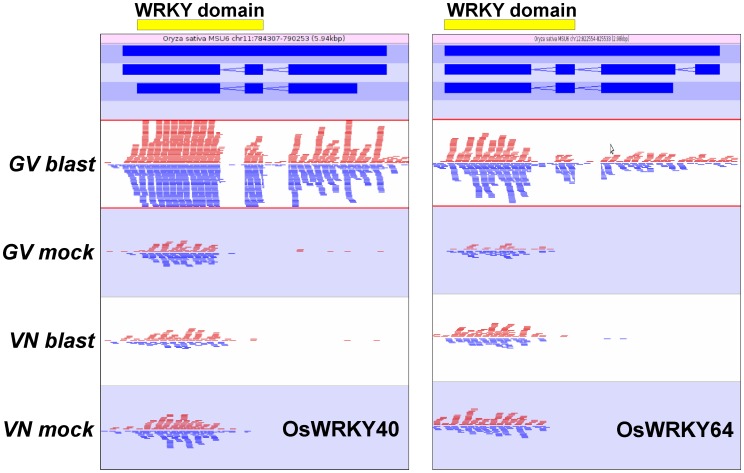
Reads mapping pattern for *OsWRKY40* and *OsWRKY64.* Expression patterns for Gigante Vercelli (GV) and Vialone Nano (VN) in mock and blast inoculation conditions are reported. Red and blue segments above and below the midline represent reads mapping to forward and reverse strands, respectively. Localizations of exons coding for the WRKY domain are indicated with yellow bars in the upper panels. Blue bars in the second panel represent gene, mRNA and coding sequence structure respectively from the upper to the lower bar.

The Cufflinks software was used to identify differential expression of alternative *WRKY* transcript isoforms. Thus, in addition to the *WRKY* genes described above, several modulated *WRKY* isoforms have been identified (Table S4) and, among them, some were associated with loci for which differential expression could not be detected at the whole-gene level. Two transcripts originating from the same transcription start site were identified for *OsWRKY62*, with both isoforms strongly induced by blast infection in GV and only one, the longest of the two isoforms, moderately induced despite being weakly expressed in VN. It was previously observed that the longest isoform contains a putative sumoylation site and a potential coiled-coil domain at the C-terminus and N-terminus respectively [50], [53]). Overexpression of this isoform hampered *Xa21*-mediated resistance to *X. oryzae* pv. *oryzae* and suppressed the activation of defense-related genes, while the second isoform did not produce clear effects on *X. oryzae* pv. *oryzae* resistance, indicating that at least one *OsWRKY62* isoforms act as a negative regulator of innate immunity in rice [53]. The observed differential regulation of the *OsWRKY62* isoforms in GV might suggest a regulatory role of the longer also in rice interaction with blast. Similarly, for *OsWRKY65*, one of the most induced *WRKY* genes in GV (105-fold; Table 3), two transcripts of differing lengths, 1,443 and 1,026 bp, and different transcription start sites were identified. Both isoforms were induced in GV only with the longer representing a novel isoform (class code = j, Table S4) not included in existing annotations. Interestingly, for *OsWRKY40* (LOC_Os11g02530, 13-fold induced in GV and not modulated in VN) three different isoforms with various transcription start sites were detected. In blast infected GV the canonical 3 exon-containing major predicted isoform (including *WRKY* domains) was transcribed, while in controls and VN infected samples only a form lacking two 5′ exons was identified (Figure 6). Expression analysis with RT-PCR and quantitative RT-PCR using primers specific for exon 1 and exon 3 of *OsWRKY40* (Figure 6) confirmed that both exons were transcribed in GV challenged with blast while in GV mock-inoculated and VN (both infected and not infected samples) the transcription of the exon 3 only was observed (data not shown). As the WRKY domain is split among the two exons of the 3′ region, in VN and control GV a protein bearing a truncated WRKY domain is likely expressed. A very similar exon organization and read mapping pattern was found also in *WRKY64* (LOC_Os12g02450, 8-fold induced in GV). In conclusion, our results highlighted that *WRKY* isoforms represent an additional strategy exploited by the blast resistant genotype GV for the fine tuning of resistance responses to blast infection.

### Blast-modulated Components of the Defence Signal Transduction Pathways

#### WAK kinases

**Figure 7:**
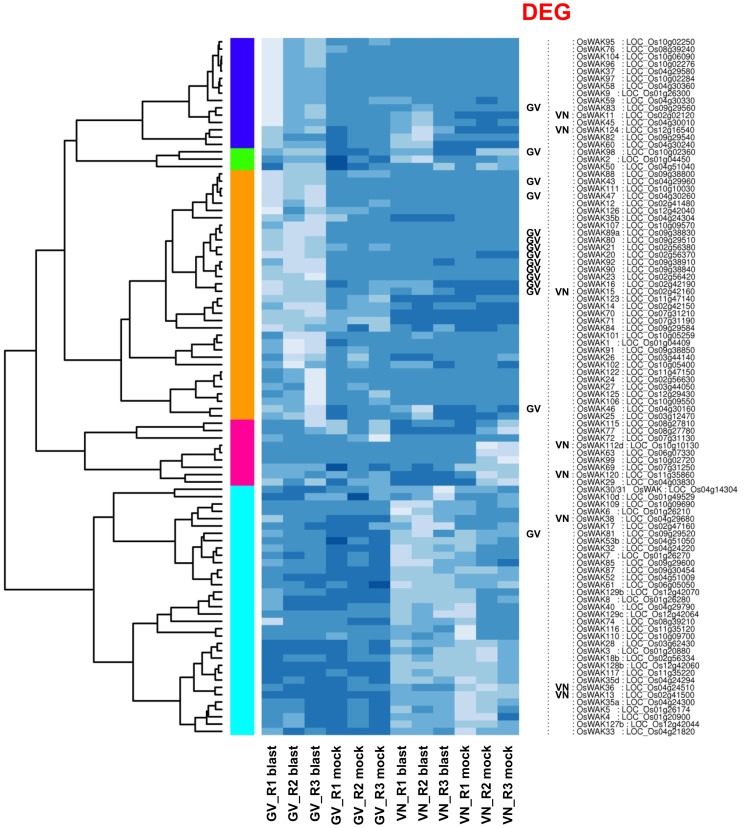
Hierarchical clustering of OsWAK gene expression. Gigante Vercelli (GV) and Vialone Nano (VN) *OsWAK* gene expression as affected by blast infection in the different treatments (blast or mock inoculated) and biological replicates (R1, R2, R3). Genes called as DEGs are indicated on the right border of the heatmap. Colored bars on the left of the heatmap mark distinct major branches in the clustering tree grouping genes with similar expression pattern. The colour scale indicates the expression value (light blue indicate higher expression value, darker blue indicates lower gene expression values). The heat map was generated with custom scripts based on heatmap.2 function as available in the ‘gplots’ Bioconductor package.

Wall Associated Kinases (WAK kinases) are cell wall-associated receptor kinases and represent a novel subfamily of plant Receptor-like kinases [54]. In MSU6.12, 130 genes are tagged with the keyword “*OsWAK*”. *WAK* genes present an extracellular epidermal growth factor-like domain and a putative intracellular serine/threonine kinase domain. Several WAKs bear a calcium-binding domain and their up-regulation in GV contributed to the enrichment of the GO term “calcium ion binding” (GO:0005509). In Arabidopsis, *WAKs* are involved in cell expansion, pathogen resistance and heavy metal stress tolerance [55], while AtWAK1 potentially acts as a receptor of oligogalacturonides elicitors [56]. WAKs link the plasma membrane to the cell wall matrix through their extracellular receptor domain, and can link perception of external signals (including pathogen attacks) to the triggering of defence mechanisms *via* their cytoplasmic kinase domains [57]. In GV, 15 *WAKs* were listed among DEGs and were all up-regulated with fold changes ranging from 2 to 30 (Table 4), while in VN only 4 *WAKs* were up-regulated and 4 down-regulated. A snapshot of the expression pattern of all rice *WAKs* highlights that 15 out of the 95 *OsWAK* genes expressed in our dataset were significantly induced by blast infection in GV and only one modulated gene was in common between susceptible and resistant genotypes (Figure 7). Despite being a very large group, rice *WAK* gene functions are poorly characterized. *OsWAK1* has recently been shown to be induced in response to blast infection and its over-expression conferred enhanced resistance to the fungus [58]. In our dataset, *OsWAK1* was strongly constitutively expressed in both GV and VN and although a slight up-regulation (1.9-fold) was observed in GV alone, this modulation was not statistically significant (Table S1). Nevertheless, several up-regulated *WAKs* clustered with *OsWAK1* in the heatmap (orange cluster in Figure 7); this observation, together with a role in blast resistance for a member of this receptor-like kinase group, could indicate a direct role in GV blast resistance for additional WAK proteins.

**Table 4:** *WAK* (Wall Associated Kinase) genes modulated in Gigante Vercelli and Vialone Nano.

Gigante Vercelli						
id	MSU 6.12 description		Mock inoculated^a^	Blast inoculated^a^	Fold change	FDR
LOC_Os09g38830	OsWAK89a - OsWAK receptor-like protein kinase		0.36	10.91	30.15	0.02
LOC_Os09g29560	OsWAK83 - OsWAK pseudogene		16.28	261.69	16.07	4.00E−003
LOC_Os04g30260	OsWAK47 - OsWAK receptor-like protein kinase		1.21	15.75	12.98	0.01
LOC_Os09g29510	OsWAK80 - OsWAK receptor-like protein kinase, expressed		3.09	36.02	11.65	2.78E−005
LOC_Os02g56420	OsWAK23 - OsWAK short gene, expressed		8.97	81.53	9.09	0.00
LOC_Os04g29960	OsWAK43 - OsWAK receptor-like protein kinase, expressed		10.75	70.60	6.57	8.16E−006
LOC_Os09g38840	OsWAK90 - OsWAK receptor-like protein kinase, expressed		34.93	198.25	5.68	9.23E−006
LOC_Os04g30160	OsWAK46 - OsWAK receptor-like protein kinase, expressed		58.60	220.55	3.76	0.01
LOC_Os02g56370	OsWAK20 - OsWAK receptor-like protein kinase, expressed		110.49	408.13	3.69	2.48E−005
LOC_Os02g56380	OsWAK21 - OsWAK receptor-like cytoplasmic kinaseOsWAK-RLCK, expressed		159.08	586.36	3.69	8.19E−006
LOC_Os02g42190	OsWAK16 - OsWAK receptor-like protein kinase		89.20	296.79	3.33	3.89E−004
LOC_Os09g29520	OsWAK81 - OsWAK receptor-like cytoplasmic kinaseOsWAK-RLCK, expressed		49.89	165.73	3.32	0.03
LOC_Os09g38910	OsWAK92 - OsWAK receptor-like protein kinase, expressed		78.23	230.60	2.95	0.04
LOC_Os10g02360	OsWAK98 - OsWAK receptor-like cytoplasmic kinaseOsWAK-RLCK, expressed		159.85	384.81	2.41	0.02
LOC_Os02g42160	OsWAK15 - OsWAK receptor-like protein kinase		104.13	231.66	2.23	0.03
**Vialone Nano**						
**id**		**MSU 6.12 description**	**Mock inoculated** a	**Blast inoculated** a	**Fold Change**	**FDR**
LOC_Os02g02120		OsWAK11 - OsWAK receptor-like protein kinase	60.76	277.01	4.56	0.02
LOC_Os12g16540		OsWAK124 - OsWAK receptor-like protein OsWAK-RLP,expressed	93.86	325.57	3.47	0.03
LOC_Os04g29680		OsWAK38 - OsWAK receptor-like protein kinase, expressed	64.48	216.66	3.36	5.78E−004
LOC_Os02g42160		OsWAK15 - OsWAK receptor-like protein kinase	34.69	98.26	2.83	0.01
LOC_Os02g41500		OsWAK13 - OsWAK receptor-like protein kinase, expressed	277.09	133.46	0.48	0.02
		OsWAK36 - OsWAK receptor-like protein kinase, expressed	191.57	64.74	0.34	2.25E−003
LOC_Os11g35860		OsWAK120 - OsWAK receptor-like protein kinase	101.62	14.87	0.15	0.04
LOC_Os10g10130		OsWAK112d - OsWAK receptor-like protein kinase,expressed	112.38	0	-Inf	0.01

a Expression values are reported as DESeq-normalized read counts.

#### Chitin elicitor sensing components

The earliest characterized in blast sensing involve the binding of chitin oligosaccharides by CEBiP (chitin elicitor binding protein; LOC_Os03g04110) and a chitin elicitor receptor kinase, OsCERK1 (LOC_Os08g42580, a LysM receptor-like kinase). Upon chitin elicitor binding, CEBiP forms hetero-oligomers with OsCERK1 which, in turn, possibly through transphosphorylation, triggers downstream signalling [59], [60]. Strikingly, *OsCERK1* knockdown lines exhibited both a strongly depressed ROS upsurge and accumulation of phytocassanes and momilactones when challenged with elicitors. However, *OsCERK1* knockdown cell lines did not completely abolish elicitor responses, raising the possibility that other *OsLysM-RLKs* may play a sensing role [60]. In our dataset, both *CEBiP* and *OsCERK1* were strongly expressed in GV and VN with a moderate induction upon infection, while additional *OsLysM-RLK* genes were induced in GV (Table 5). Notably, *OsLYK4* was strongly constitutively transcribed (at levels higher than *OsCERK1*) and 2.34-fold induced in GV, despite not being included in DEGs. Two receptors not identified in previous studies, *OsLYK2* and *OsLysM-RLK7*, were up-regulated and listed among DEGs in GV but not in VN. These OsLysM-RLKs may contribute part of the GV-specific resistance responses and could represent the previously unidentified (or GV-specifically induced) OsLysM-RLKs responsible for the residual chitin sensing in *OsCERK1* knockdown cell lines. The expression values of additional, less-characterized LYS-M domain containing genes are listed in Table S5 (IPR018392; Peptidoglycan-binding lysin domain).

**Table 5:** Compared expression of known and putative elicitor sensing/signalling components in Gigante Vercelli (GV) and Vialone Nano (VN).

ID MSU 6.12	Gene name/synonims	MSU 6.12 description		Mock inoculated^a^	Blast inoculated^a^	Fold change	FDR	Listed among DEGs
LOC_Os03g04110	CEBiP	lysM domain-containing GPI-anchored protein precursor, putative, expressed	GV	1032.09	1830.44	1.77	0.5	NO
			VN	614.69	1429.76	2.33	5.96E−004	YES
LOC_Os08g42580	OsCERK1(OsLysM-RLK9)	protein kinase domaincontaining protein, expressed	GV	710.33	1103.54	1.55	0.52	NO
			VN	973.37	969.67	1.00	1.00	NO
LOC_Os02g09960	OsLYK4(OsLysM-RLK1)	LYK8, putative, expressed	GV	759.72	1775.77	2.34	0.25	NO
			VN	196.42	486.75	2.48	0.19	NO
LOC_Os06g41980	OsLYK2(OsLysM-RLK5)	LYK8, putative, expressed	GV	15.9	73.67	4.63	0.01	YES
			VN	16.75	53.55	3.2	0.16	NO
LOC_Os11g35330	OsLysM-RLK7	LYK, putative, expressed	GV	19.93	145.12	7.28	0.03	YES
			VN	15.91	43.17	2.71	0.56	NO

a Expression values are reported as DESeq-normalized read counts.

#### MAP kinases

Mitogen-activated protein kinase (MAPK) cascades represent candidates for downstream signalling processes, as they exert similar roles in a variety of signalling contexts downstream of receptor kinases, including the biosynthesis of the phytoalexin camalexin in Arabidopsis [34]. MAPK cascades consist of evolutionarily conserved kinase signaling modules involving at least three kinases: a MAPK, a MAPK kinase (MAPKK) and a MAPKK kinase (MAPKKK) [61]. Recently, a MAPK cascade involving the MAPKK OsMKK4 and the MAPKs OsMPK3 and OsMPK6 was shown to transduce chitin elicitor signal in rice to defense responses including biosynthesis of diterpenoid phytoalexins [62]. In partial agreement with these results, in our dataset the *OsMPK3* gene was significantly induced in GV (listed as DEG with a 2.35 fold change), while *OsMKK4*, despite the strong constitutive expression in GV, was listed as a DEG only in VN (Table S6). Finally, a strong constitutive expression and a slight but not significant induction of *OsMPK6* was observed in both rice genotypes (Table S6).

Despite recent advances in our understanding of this MAPK cascade, much less is known of the upstream MAPKKK (MAP3K) which actually trigger the cascade [44]. *MAP3K.18* and *MAP3K.19* (LOC_Os05g46750 and LOC_Os05g46760, respectively) were up-regulated and called as DEGs in GV only (Table S6). Interestingly, several rice microarray experiments indicate that these *MAP3Ks* should be upstream of the *MAPK* and *MAPKK* genes in different developmental stages and in response to various biotic and abiotic stresses [63]. Transcriptional induction of *MAP3K* in GV could therefore drive the activation of several downstream *MAPKKs*, whose genes are not modulated transcriptionally level by the infection process.

**Figure 8:**
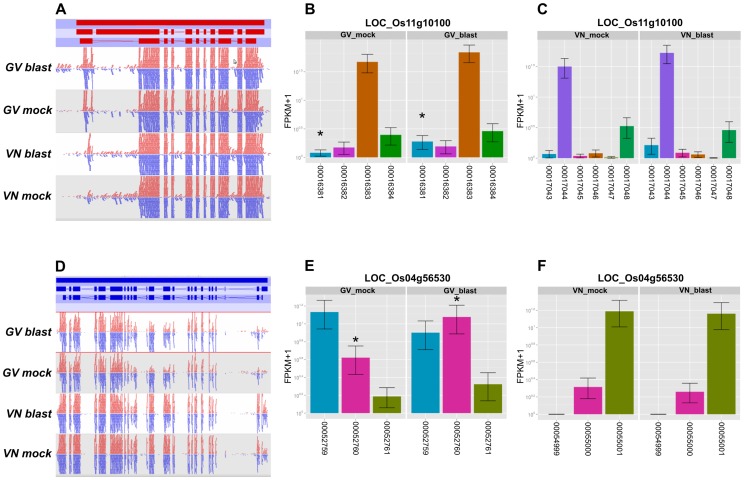
*MAP3K* isoform analysis. **A**: *MAP3K*.3 (LOC_Os11g10100 ) read mapping pattern in mock and blast inoculation conditions in Gigante Vercelli (GV) and Vialone Nano (VN) (red bars in the upper panel represent gene, mRNA and coding sequence structure respectively from the upper to the lower bar); red and blue segments above and below the midline represent reads mapping to forward and reverse strands, respectively. **B**: transcript isoforms and associated Cufflinks expression values in GV; **C**: transcript isoforms and associated Cufflinks expression values in VN. **D**: *MAP3K.1* (LOC_Os04g56530) reads mapping pattern in mock and blast inoculation conditions in GV and VN (blue bars in the upper panel represent gene, mRNA and coding sequence structure respectively from the upper to the lower bar); red and blue segments above and below the midline represent reads mapping to forward and reverse strands, respectively. **E**: transcript isoforms and associated Cufflinks expression values in GV; **F**: transcript isoforms and associated cufflink expression values in VN. Differentially expressed isoforms are indicated by asterisks.

Finally, isoform analysis (Table S7) revealed that further *MAP3Ks* not significantly modulated at transcript level were however modulated at the levels of alternative splicing/alternative transcripts in GV but not in VN. The *MAP3K.3* (LOC_Os11g10100) is predicted to produce 4 and 6 isoforms in GV and VN, respectively. One of the 4 GV isoforms (00016381) was up-regulated in GV only (fold change 8.54, FDR 0.03), while none of the 6 VN isoforms was modulated upon infection (Figure 8A–C) Similarly, also *MAP3K.1* (LOC_Os04g56530) was predicted to produce an up-regulated (fold change 5.95; FDR 0.000) isoform in GV only (Figure 8D–F). Once again, as pointed out for *WRKY* gene expression analyses, RNA-Seq approach allowed to highlight the additional level of gene expression regulation represented by the blast infection-dependent modulation of different transcript isoforms.

### Transcriptional Activation of Downstream Defence Genes

**Figure 9:**
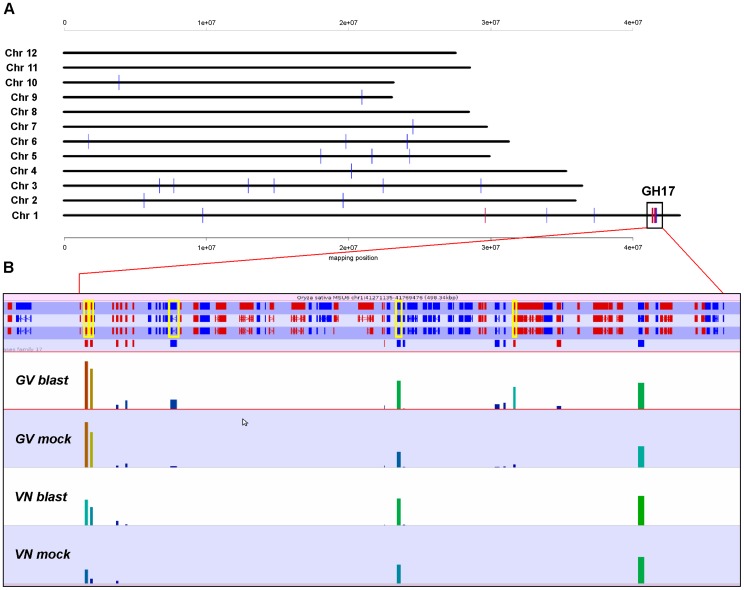
Analysis of differential gene expression for glycosyl hydrolases gene family 17. **A**: Chromosome localizations of glycosyl hydrolase family 17 (GH17) genes. Blue bars represent all GH17 genes. Red bars indicate significantly up-regulated genes in Gigante Vercelli (GV) upon blast infection; physical distances for each rice chromosome are also indicated (mapping position values in millions bp). **B**: Detail of the chromosome 1 region boxed in panel A. The 350 kb region containing 14 GH17 genes is magnified and GH17 genes are indicated with a red (forward) or blue (reverse) mark below genes. Bars with heights proportional to read counts in GV and Vialone Nano (VN) are shown below genes. The five GH17 genes significantly up-regulated in GV (DESeq calls) are boxed in yellow.

The GV-specific enrichment of the GO term “defence response to fungus” (GO:0050832), reflects induction of additional defence genes in GV including the two chitinase genes *CHIT17* and *CHIT7* (LOC_Os05g33130 and LOC_Os06g51050; Chitinase family protein precursor) showing 37- and 8-fold induction, respectively (Table 6). Both, *CHIT17* and *CHIT7*, are class I chitinases with glycosyl hydrolase family 19 (GH19) catalytic domains and chitin binding domains [64]. Class I vacuolar chitinases are reputed to exert effective defence roles against fungi and to participate in the plant-pathogen co-evolution process; the high ratios of non-synonymous to synonymous substitutions rates in the coding regions indicating positively selected amino acid replacements [64], [65]. Furthermore, significant and simultaneous up-regulation of six of the nine endo-β-1,3-glucanases (glycosyl hydrolases family 17, hereafter GH17; Table 2) listed in GO group “defence response to fungus, incompatible interaction” (GO:0009817) was observed, while only non-significant, weak induction trends were observed in VN. Notably, five of the six GV up-regulated GH17 genes are clustered within a 350 kbp region on chromosome 1 (Figure 9). Similarly to GH19 chitinases described above, *GH17* genes appear to be subjected to rapid evolution as shown by the observed high rate of non-synonymous substitutions [66]. In addition to the direct protective role in fungal cell wall degradation exerted in combination with chitinases, one or more *GH17* gene members may contribute to elicitors generation by oligosaccharide production [67].

**Table 6:** Comparison of expression values for significantly up-regulated genes belonging to the GO group “defence response to fungus” (GO:0050832) in Gigante Vercelli and Vialone Nano.

Gigante Vercelli								
id	MSU 6.12 description		Mockinocuated^a^	Blastinoculated^a^	FoldChange	FDR	Listedamong DEGs	
LOC_Os03g08410	flavin-containing monooxygenasefamily protein, putative, expressed		0.72	34.41	47.54	2.22E−007	YES	
LOC_Os03g13180	peroxidase precursor, putative, expressed		1.84	17.29	9.38	0.020	YES	
LOC_Os05g33130	CHIT17 - Chitinase family protein precursor, expressed		4.70	176.21	37.49	1.45E−004	YES	
LOC_Os06g51050	CHIT7 - Chitinase family proteinprecursor, expressed		66.82	537.09	8.04	3.15E−007	YES	
LOC_Os08g17680	stromal cell-derived factor 2-like protein precursor,putative, expressed		1294.12	2685.46	2.08	0.030	YES	
LOC_Os11g37950	WIP3 - Wound-induced protein precursor,expressed		54.82	463.30	8.45	5.97E−013	YES	
LOC_Os11g37960	WIP4 - Wound-induced protein precursor,expressed		185.27	973.62	5.26	7.97E−011	YES	
**Vialone Nano** b								
**id**		**MSU 6.12 description**	**Mock** **inoculated** a	**Blast** **inoculated** a	**Fold** **Change**	**FDR**		**Listed** **among DEGs**
LOC_Os05g33130		CHIT17 - Chitinase family protein precursor, expressed	1.26	2.77	2.19	0.990		NO
LOC_Os06g51050		CHIT7 - Chitinase family protein precursor, expressed	45.49	230.12	5.06	0.460		NO
LOC_Os08g17680		stromal cell-derived factor 2-like protein precursor,putative, expressed	1427.62	2013.28	1.41	0.700		NO
LOC_Os11g37950		WIP3 - Wound-induced protein precursor, expressed	28.07	404.86	14.42	0.010		YES
LOC_Os11g37960		WIP4 - Wound-induced protein precursor, expressed	74.53	548.98	7.37	0.110		NO

a Expression values are reported as DESeq-normalized read counts.

b Lack in the VN list of the corresponding GV induced gene denotes expression below the expression cutoff.

Other genes belonging to GO group 0050832 and up-regulated in GV only (Table 6) include a flavin-containing monooxygenase (LOC_Os03g08410) which was strongly induced (47-fold) in GV (below expression threshold in VN), a further flavin-containing monooxygenase (LOC_Os04g14690) that was 3.9–fold induced in GV and several other monooxygenase genes showing induction in GV despite not reaching an FDR <0.05 (Table S1).

### Time Course Analysis and Validation of Selected DEGs

**Figure 10:**
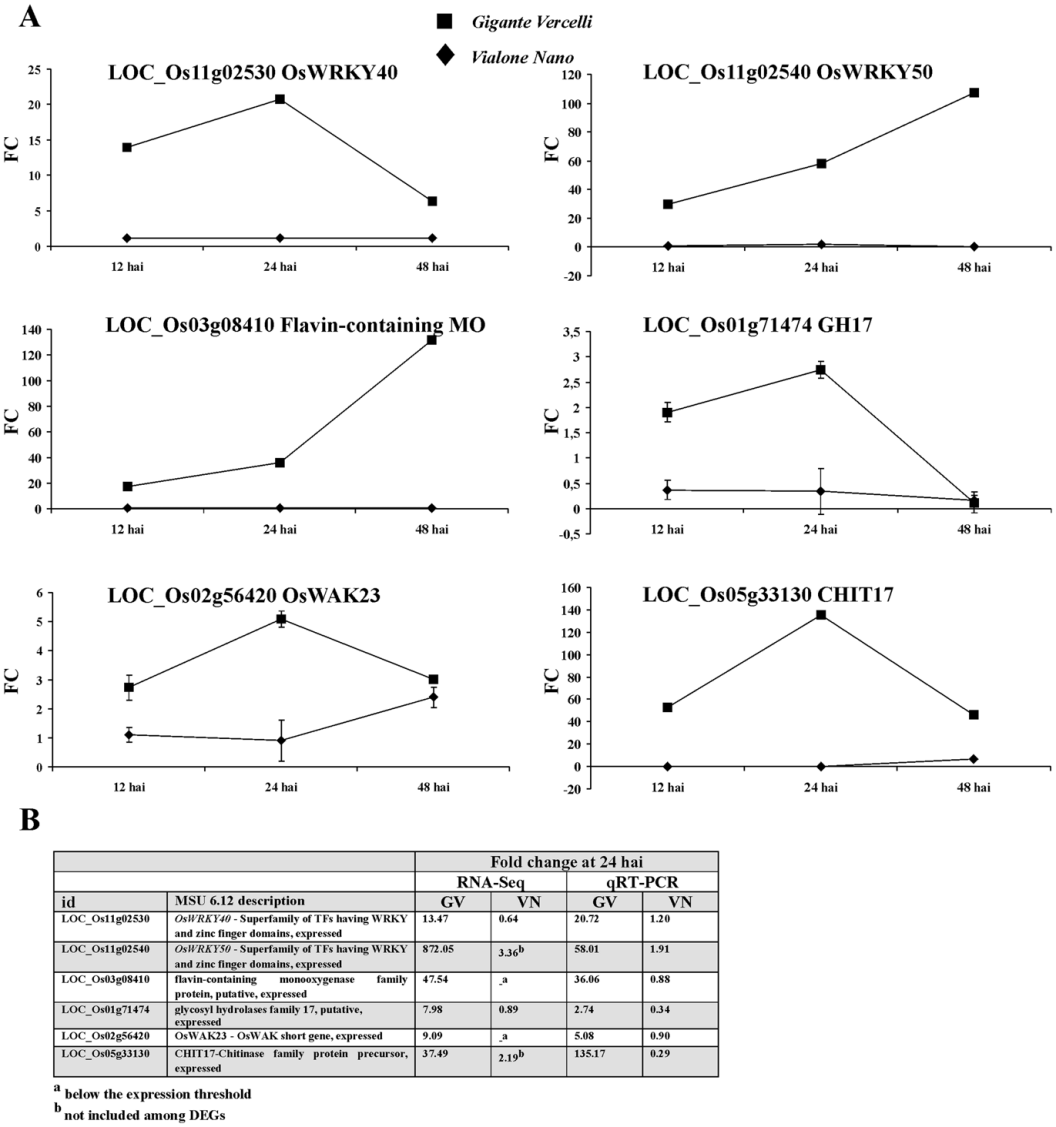
Time course expression analysis and data validation for six selected differentially expressed genes (DEGs). **A**: Quantitative RT-PCR at 12, 24 and 48 hours after pathogen inoculation (hai) for six selected DEGs in Gigante Vercelli and Vialone Nano. Values are expressed as fold changes of transcript levels in the inoculated samples with respect to the transcript levels in mock-inoculated rice leaves. Error bars represent SD across all RT-PCR replicates (three from each of two independent inoculations); when error bars are not visible is because their extent is shadowed by filled squares and rhombus indicating the rice genotypes. **B**: Comparisons of transcripts fold changes as detected by RNA-Seq or qRT-PCR expression analysis techniques for the six selected genes in Gigante Vercelli (GV) and Vialone Nano (VN) rice genotypes.

Gene expression changes for six selected genes identified as DEGs during the RNA-Seq experiments were monitored at three inoculation time points (12, 24 and 48 hai) by means of quantitative real-time PCR (qRT-PCR). In GV, transcript levels of *OsWRKY50* and flavin-containing monooxygenase showed a steady increase in response to blast inoculation with fold changes values of about 20 already at 12 hai (Figure 10A). Blast responsiveness of the other four genes (*OsWRKY40*, *GH17*, *OsWAK23* and *CHIT17*) was characterized by a similar trend of transcripts accumulation, with an increased expression from 12 to 24 hai followed by a decrease at 48 hai. At this last time point, with the exclusion of *GH17*, the gene expression in the inoculated samples was however higher than the correspondent mock inoculated counterpart, with fold changes values ranging from about 3 (*OsWAK23*) to about 40 (*CHIT17*). In VN, with the exception of *OsWAK23*, for which a blast responsiveness was detected at 48 hai, no significant induction was observed in the qRT-PCR experiments for the tested genes.

The time course experiment clearly support the choice of the sample at 24 hai as the most suitable for an extensive transcriptomic analysis, furthermore the comparison between RNA-Seq and qRT-PCR fold changes data at 24 hai revealed a substantial agreement in the extent of the blast-induced variations for transcripts accumulation in the six tested genes (Figure 10B).

### Expression Analysis of Putative Disease Resistance Genes

**Figure 11:**
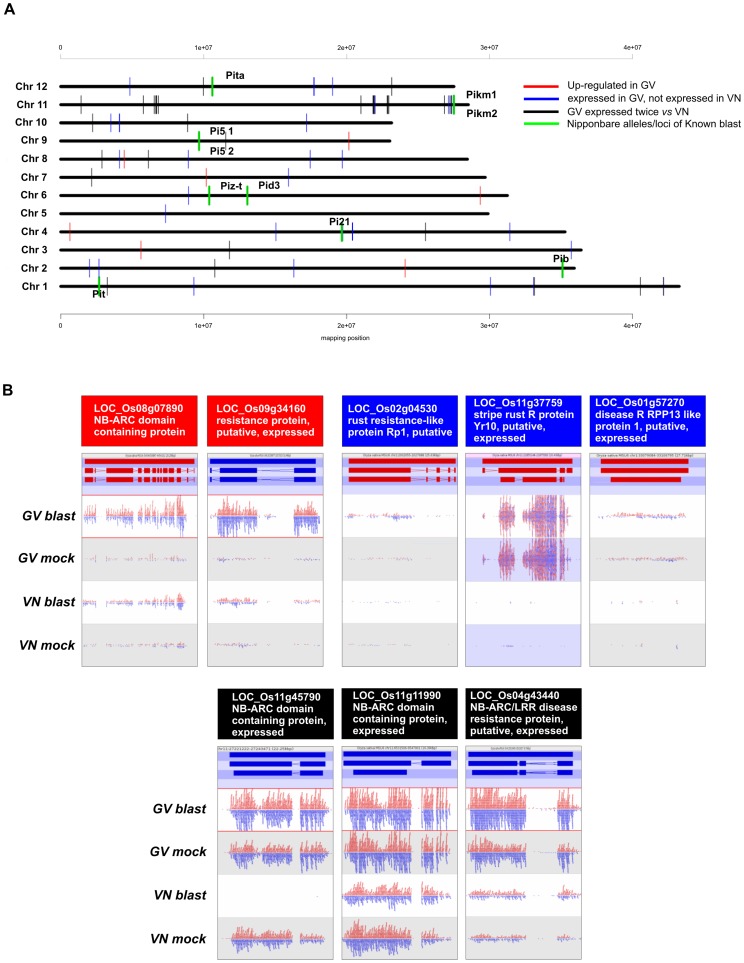
Expression analysis of Resistance Genes Analogs (RGAs) candidates for Gigante Vercelli (GV) resistance functions. **A**: Chromosome localization of candidate resistance (*R*) genes. Red bars: GV-specific induced RGAs called as DEGs; blue bars: RGAs expressed in GV only (i.e. expressed in GV, not expressed in Vialone Nano -VN-, regardless to infection responsiveness); black bars: RGAs with at least twofold expression ratio among infected GV and infected VN. Nipponbare loci corresponding to known blast *R* genes are indicated with green, thick bars; physical distances for each rice chromosome are also indicated (mapping position values in millions bp). **B**: Read mapping patterns in mock and blast inoculation conditions of selected RGAs from the three groups listed above for Gigante Vercelli (GV) and Vialone Nano (VN). Each group is color-coded as in legend of panel A. Red and blue bars in the upper panel represent gene, mRNA and coding sequence structure respectively from the upper to the lower bar. Red and blue segments above and below the midlines represent reads mapping to forward and reverse strands, respectively.

Identification of blast resistance (*R*) genes is a key objective in rice breeding and more than 80 genes encoding resistance to various combinations of blast races are known [68], [9]. To date, 13 major blast *R* genes have been cloned, 12 of which belong to the large NB-LRR gene family of disease resistance genes [10]. To pinpoint genes candidates for GV durable blast resistance, a similarity search for genes showing homology to known *R* genes was conducted on our dataset. Despite the fact that transcriptional modulation is not required for a *R* gene to be considered a candidate *R* gene (transcription of most *R* genes is not responsive to pathogen infection; [69]), a moderate level of pathogen responsiveness in terms of gene transcription (e.g. 30% induction over controls) has been reported for several active *R* genes (e.g. *Xa1, Xa2*7; [70]). Such responsiveness was higher in the case of the blast resistance *Pib* gene [71] and the expression of some *R* genes may be detectable only upon pathogen challenge [69]. Based on the above criteria, biomaRt queries (MSU release 6.12) were used to retrieve all Nipponbare genes tagged by the INTERPRO domain IPR000767 (“disease resistance protein”). The entire list (521 genes identified in GV and VN) is reported in Table S8 with accompanying expression values (DESeq normalized expression values) or not assigned values (NA in case of genes with RPKM <0.1). In a few cases where the reported MSU 6.12 gene descriptions for the IPR000767 “disease resistance proteins” did not support a putative disease resistance role, due to different database annotation procedures, we added in parentheses an alternative description of the best blast hit supporting such a putative role (Table S8). In order to identify resistance gene analogs (RGAs) candidate for the GV resistance, the list of 521 genes was filtered according to the following criteria: a) transcriptional induction by the blast pathogen in GV only (i.e. GV-specific induced RGA genes called as DEGs); b) genes expressed in GV only (i.e. expressed in GV, not expressed in VN, regardless of infection responsiveness); and c) expression ratio among infected GV and infected VN of at least two, regardless of infection responsiveness (Table S9; redundancies with previous entries were removed). A total of 75 candidate RGAs (8, 32 and 35 according to the a), b) or c) above mentioned criteria, respectively) were localized on the 12 rice chromosomes (Figure 11A). The analysis of possible co-localization between the position in the Nipponbare genome of alleles or loci of known blast resistance genes (e.g. *Pit*, *Pib*, *Pi21*, *Pid3*, *Pi*-*zt*, *Pi-5*, *Pi-km*, *Pi-ta*) and the identified 75 RGAs candidates highlighted proximity relationships on rice chromosome 4 between an RGA expressed in GV only (LOC_Os04g32940) and the *pi21* gene (LOC_Os04g32850; [17]) underlying a blast resistance QTL and on chromosome 11 between a cluster of differentially expressed RGAs and the *Pi-km1/*2 blast resistance locus (Figure 11A). Read mapping patterns for selected genes belonging to the three classes of candidate RGAs (significantly up-regulated only in GV, expressed in GV only and induced more than two times in GV) is reported in Figure 11B. A detailed analysis of the results highlights that, for some RGAs (LOC_Os11g45790; LOC_Os11g11990), expression in VN is abolished or reduced by blast infection while no differences or some up-regulation were observed for GV (Figure 11B). This result supports a possible suppression of defence responses by pathogen effectors in the susceptible genotype, an observation that is in agreement with previous reports in which fungal pathogens secrete protein effectors capable of suppressing resistance gene-based and basal defence responses in compatible interactions [72]–[75].

The 75 RGAs identified represent possible candidate genes for GV blast resistance. The comparison of their map positions with those of the genetic loci explaining GV resistance (mapping work currently in progress) will both allow identification of co-positional relationships between candidates RGAs with resistance loci and considerably restrict the number of candidates assisting in the identification of the genuine GV resistance gene(s).

### Co-regulation Analysis of GV Blast-modulated Genes

**Figure 12:**
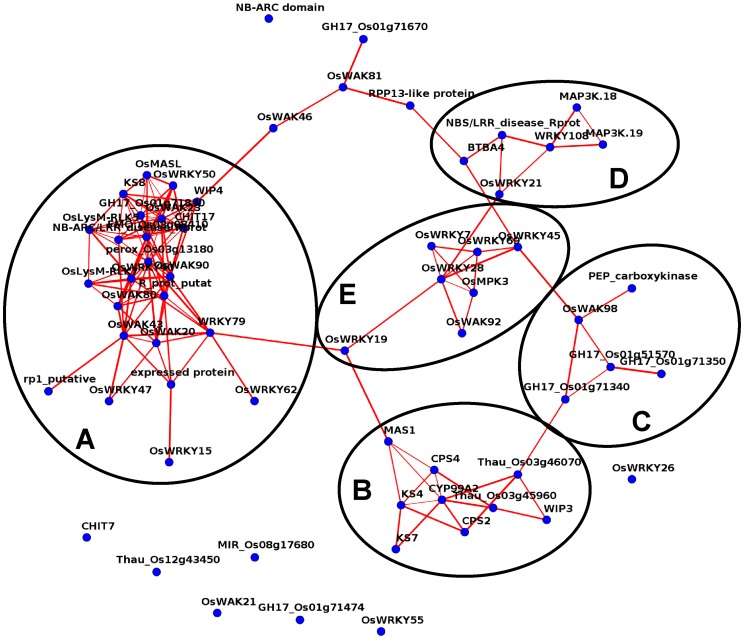
Co-regulatory networks of genes differentially modulated in response to blast inoculation in Gigante Vercelli (FDR <0.05). Co-regulation analysis was based on the calculation of pairwise Pearson correlation of logarithmic expression values. Five sub-networks, indicated with letters from A to E, were identified on the basis of pairwise Pearson Correlation coefficient with a threshold value of 0.6. The thickness of the edges is proportional to the Pearson Coefficient value and graphical visualization of the network is based on Fruchterman-Reingold layout algorithm.

Co-regulation analysis of a sub-set of GV DEGs for which a detailed dissection was conducted in the present study, accounting for a total of 63 genes (Table S10), was carried out by calculating the pairwise Pearson correlation using the Rice Expression Matrix (REM) and RI-REG software [76]. The REM was based on Affymetrix GeneChip® Rice Genome Array data including all publicly available Affymetrix experiments in a large range of conditions (see Materials and Methods section) and RI-REG was applied to verify whether genes listed as DEGs in this study were effectively modulated, and eventually co-regulated, also when data were obtained with a different expression profiling technology. We observed that 55 out of the tested 63 genes were present in a large network, confirming our findings obtained by RNA-Seq analysis (Figure 12). In addition, in this large network at least half of the genes were linked with multiple connections suggesting tight relationships between the GV blast-infection-related DEGs. In particular, one big and four small sub-networks were identified, representing clusters of genes which might have specific functions in cooperating together, is agreement with a putative coordinated role of these genes in GV resistance responses. The big sub-network A included genes belonging to all defence pathways identified in the present work and activated during GV resistance response to blast. In this sub-network are genes involved in perception (*OsWAK*, putative disease resistance protein rp1, NB-ARC/LRR disease resistance protein, putative R protein), signalling (several *OsWRKYs* and *LYS-RLK* genes) and genes participating to downstream defence pathways induced to block pathogen invasion, such as the GO group “defence response to fungus” genes (GO:0050832: *chitinase17*, *peroxidase*, *FMO*, *WIP4*) and genes involved in phytoalexin synthesis (*OsMASL*, *KS8*). *OsWRKY47*, *15*, *42*, *79*, *40* and *OsWRKY50* are present in this sub-network, again supporting the crucial role of *OsWRKYs* in fine tuning blast response strategies. A smaller sub-network B included genes specifically involved in phytoalexin biosynthesis, while the small sub-network C mainly grouped genes induced during preliminary stages of the perception and downstream defence responses (*WAKs* and *GH17*). Genes involved in signalling (*MAPK* and *OsWRKY*) are present in sub-network D, which could be potentially involved in specific interactions, as suggested by the presence of *BTB4*, a member of the Bric-a-Brac/Tramtrack/Broad Complex (*BTB*) gene family. BTB proteins direct the selective ubiquitination of proteins following their assembly into Cullin3-based ubiquitin ligases, allowing selective breakdown of short-lived regulatory proteins, which is a key feature of many signal transduction pathways [77]. The sub-network E in the centre of the large network, is linked to all the above mentioned sub-clusters and included only signalling-related genes, indicating that it could potentially play a central role as master regulator for the other sub-networks. The presence of several sub-networks linked together within the large main cluster might suggest that GV resistance response involve a fine coordinated activation and regulation of several pathways that collaborate to confer blast resistance.

### Conclusions

RNA-seq was employed in this study to exploit several advantages of this technique over microarray-based expression profiling; in particular RNA-seq is not dependent from existing genome sequence knowledge, shows reduced background levels, offers higher dynamic range of detection and allows for dissection of expression down to the transcript isoform level. [78].

Whole transcriptome sequence data from the blast-resistant rice genotype Gigante Vercelli (GV) and the susceptible Vialone Nano (VN) at 24 hpi, an inoculation time point that overlaps the early induction of resistance gene-dependent defence reactions in blast resistant rice genotypes, were compared. Plasma membrane damage occurring in blast-resistant rice genotypes is slightly deferred with respect to 24 hpi, indicating that crucial events responsible for halting blast infection should be analyzed within this temporal frame. The analysis provided an unprecedented global overview of the defence processes differentiating a durable resistant from a susceptible response to blast infection that could ultimately be related to the resistant phenotype. Differential responses included a prompt induction in GV of DPB genes producing phytocassane, momilactone and oryzalexin S phytoalexins. As prompt accumulation of phytocassanes and momilactones alone is known to effectively limit *M. oryzae* spreading [39], GV resistance most likely exploits the available spectrum of defensive diterpene phytoalexin compounds (i.e. including oryzalexin S). Simultaneously, genes encoding well-characterized antifungal enzymes encompassing class I chitinases and glycosyl hydrolases were strongly up-regulated. These classes of defence response genes were missing or weakly paralleled in VN. Differences in the induction of this battery of early defence responses between resistant and susceptible genotypes appears to be coordinated, on the basis of our expression data, by discrete members of signal transduction pathways components that include WRKY transcription factors, WAK family of elicitor receptors, chitin-oligosaccharide sensing machinery and mitogen-activated protein kinase (MAPK) cascades. Adoption of the RNA-Seq approach also allowed to uncover an additional level of regulation in the resistant blast response consisting in the modulation of discrete isoforms of *WRKY* and *MAP3K* transcripts and this response was observed in GV but not in VN. Differential transcript levels both for downstream effector genes and genes involved in regulatory functions in the susceptible genotype ranged from lower gene expression to abolishment of gene transcription. This behaviour might be ascribed to the lack of effective pathogen-sensing mechanisms and, possibly, to the effect of pathogen effectors suppressing VN basal defence responses. Finally, by utilizing expression pattern-based selection criteria, a list of RGAs representing candidates for GV resistance functions were identified and assigned to rice chromosomes. These will represent useful targets in assisting the map-based isolation of the genuine GV resistance gene(s). Most of the genes analysed in detail in this work were included in a co-regulatory network that supports a coordinated regulation of GV resistant response to blast infection. A sub-network containing only signalling-related genes was identified, suggesting that genes included might play central roles as master regulators for the other sub-networks. The data presented here should help to further clarify the mechanistic events underlying successful and durable resistance against *M. oryzae* and functional studies on the relevant genes are expected to prove useful in the improvement of blast susceptible rice germplasm.

## Materials and Methods

### Plant Material and Blast Infection Procedures

The experiments were carried out with *Oryza sativa* L. cvs. Gigante Vercelli and Vialone Nano. Gigante Vercelli is an old Italian variety released in 1946 and is characterized by a durable resistance to blast, while Vialone nano was released in 1937 and is highly susceptible to rice blast. Rice seeds were germinated in the dark at 28°C and distributed into pots with three biological replicates each for the control (mock inoculated) and blast inoculated conditions. Fifteen plantlets for each biological replicate were grown for 20 days at 28°C in a growth chamber. *Magnaporthe oryzae* inoculation was performed using three Italian blast isolates It2, It3 and It10 from the CIRAD collection (Montpellier, France), and previously described by [29]. These strains were cultured as previously described [76]. Conidia were harvested from plates by rinsing with sterile distilled water and filtering through two layers of gauze and a 50,000 conidia/ml suspension with 0.5% gelatine was used for inoculation. The suspension solution without conidia was used to spray the control plants (mock inoculation). Rice plants were incubated for 16 h at 28°C and 99.9% relative humidity in the dark and then transferred back to the previous growth conditions for eight hours before leaves sampling. After seven days, macroscopic phenotypes (necrosis, lesions) on rice leaves were observed on the susceptible genotype Vialone Nano acting as control of the inoculation procedure.

### Illumina GAIIx Sequencing

Total RNA was isolated using Trizol reagent (Invitrogen) according to the manufacturer’s instructions. Five micrograms of total RNA were subjected to library preparation using the “TruSeq RNA sample preparation kit (FC-122-1001)” following the manufacturer’s instructions. Libraries were amplified by 15 cycles of PCR and then purified and size selected for an average size of 300 bp by a 2% low range ultra agarose gel (BIO-RAD). RNA quality and library concentration and size were assayed on a 2100 Bioanalyzer (Agilent). Libraries were paired end sequenced for 37 bp on each end on an Illumina Genome Analyser (GAIIx). Paired end sequencing was employed to improve specificity of mapping over the reference genome due to the relatively small length of end sequencing (37 bp), which may increase risk of the same read mapping to multiple sites. Samples were run using one lane per library.

### Quantitative RT-PCR Analysis

Quantitative RT-PCR was performed using a One Step Real Time PCR System (Applied Biosystems, Foster City, CA, USA) and 100 ng of total RNA per reaction. Primers were designed using Primer3 software (http://frodo.wi.mit.edu/primer3/) (Table S11) and primers specificity was evaluated by blasting primer sequences against the NCBI database. The rice ubiquitin-conjugating enzyme E2 constitutively expressed gene [79] was used as reference gene for normalization. PCR amplifications were performed in 25 µl of final volumes containing 2× QuantiFast SYBR® Green Master Mix (Qiagen, Hiden, Germany) and including ROX™ as passive reference dye, 400 µM each primer and 0.25 U/µl Multiscribe™ Reverse Transcriptase (Applied Biosystems). Three technical replicates for each of two biological replicates were performed. Relative gene expression was calculated using the 2^−ΔΔCt^ method [80].

### Bioinformatic Methods

#### Mapping of illumina reads

The CASAVA pipelines 1.7 and 1.8 were employed for data processing and basecalling for GV and VN, respectively. As the quality scores encodings for FASTQ files produced by the two pipelines are different (ASCII code +64 *vs* ASCII code +33, for CASAVA 1.7 and 1.8, respectively) downstream applications (e.g. Bowtie/TopHat) were set accordingly. In both cases, only reads passing Illumina filters reads were kept. Read quality was further assessed by fastQC application and reads containing contaminant primer/adapters and long stretches of poor quality bases were removed as necessary.

The spliced read mapper TopHat version 1.4.1 was used to map reads to Nipponbare (MSU release 6.12) genome setting a minimum intron length of 30 considering rice small intron size [81]. Mapped paired end read were deduplicated with Picard 1.63 and read counts were collected with HTSeq version 0.5.3 (http://www-huber.embl.de/users/anders/HTSeq) in the paired end and ‘union’ mode using *Oryza sativa* MSU 6.12 GTF file.

#### DEG calling

Differentially expressed genes were called *via* DESeq package [82] (version 1.6.0), an R package implementing a model based on negative binomial distribution which was developed with special attention to cope with biological variance [82]. The software was run under R release 2.14. In order to define the set of expressed genes, raw read counts as obtained by HTSeq application were normalized to RPKM (Reads per Kilobase per Million) and genes above the 0.1 RPKM threshold were considered expressed. A combination of fitted versus per-gene dispersion values was implemented as suggested by the default DESeq setting. Separate instances of DESeq CountDataSet objects were created for GV and VN to compute DEG (FDR < = 0.05).

#### GO enrichment analyses

For goseq analyses, as rice database are not yet covered by Goseq, gene lengths were retrieved with BiomaRt queries (*Oryza sativa* MSU6.12) out of rice Nipponbare cDNA and median length for each rice locus were obtained parsing with R custom scripts. An FDR cutoff of 0.05 was used for GO enrichments. Gene ontology terms for CC, MF and BP were similarly retrieved with BiomaRT queries.

#### Isoform analysis

Cufflinks version 1.3 was employed for isoform analysis. Picard-deduplicated BAM alignment files from TopHat were employed as cufflinks input. No GTF was provided to cufflinks in order to detect novel transcripts. Cuffmerge module was run with cufflinks-produced-GTF files in combination with MSU6.12 GTF file in order to facilitate comparison and detection of novel transcripts. Cuffdiff was employed for calculating significant differences among isoforms expression and was run with upper quartile normalization option to improve differential expression calls for less abundant genes and transcripts [83], [84].

#### Co-regulation analysis

The correlation analysis was performed as previously described [76]. Briefly, the Visual C++ program (RI-REG) was applied to analyse gene co-regulation based on Pearson correlation coefficient [85] for GV DEGs analysed in the present study (listed in Table S10). The RI-REG software builds a matrix of the gene list with all calculated reciprocal Pearson correlation coefficient. Pearson coefficient was calculated on Rice Expression Matrix (REM), consisting all publicly available Affymetrix experiments (749, as of October 2010) from platform GPL2025 in Gene Expression Omnibus (GEO; http://www.ncbi.nlm.nih.gov/geo/). Calculation of Pearson coefficient was based on logarithm values of the data. The GV DEGs network was obtained filtering pairwise Pearson correlation values of 0.6 or higher from the complete matrix. Intensity of arrow colours are proportional to the coefficient between each pair of genes. Graphical representations of gene networks were produced using a force-based layout algorithm of Fruchterman-Reingold available in igraph library (http://igraph.sourceforge.net; [86]).

#### Miscellaneous bioinformatic methods

R (version 2.14 and 2.15) and Python (version 2.6) programming languages were used for custom scripts. R packages were mainly obtained from Bioconductor project (http://www.bioconductor.org/). Hierarchical clustering and heatmaps and matrix distances for sample clustering were computed over Spearman correlations (a non-parametric measure of statistical dependence between two variables) using R functions.

Gene expression values for Hierarchical clustering were obtained by extracting, for each biological replicate, normalized read counts from DESeq CountDataSet instances. No reordering of samples in heatmaps was made for the sake of clarity. Matrix distances for expression heatmaps were computed over Pearson correlations of gene expression values by means of heatmap.2 function (gplots version 2.10.1 R package).

Samtools utilities [87] were used for merging of SAM files for various purposes (e.g. merging of biological replicates to simplify Seqmonk read mapping visualization). For visualization of read mapping, SAM/BAM files were loaded on Seqmonk application (http://www.bioinformatics.babraham.ac.uk/projects/seqmonk/) in the paired-end mode and discarding duplicate reads. Expression barplots of cufflinks-predicted isoforms were generated with cummeRbund R package version 1.0.0. Unless otherwise stated, chromosome maps and other graphical outputs were generated with custom R and Python scripts.

Rice *WRKY* genes in the present study are named according to the CGSNL nomenclature [88].

## Supporting Information

Figure S1 **Spearman correlations among GV and VN samples.** Panel A: Pairwise Spearman correlation (a non-parametric measure of statistical dependence between two variables) coefficients of the expression values in the different GV and VN treatments (blast or mock inoculated) and biological replicates (R1, R2, R3). Panel B: heatmap of the Spearman correlation coefficient for the expression values in the different GV and VN treatments (blast or mock inoculated) and biological replicates (R1, R2, R3). The color scale indicates the degree of correlation (white-yellow, low correlation; orange-red, strong correlation).
(TIF)

Figure S2 **DESeq dispersion values plotted against means for GV (A) and VN (B).** Empirical (black dots) and fitted (red lines) values are shown.
(TIF)

Table S1 **Expression values reported as DESeq-normalized read counts and fold changes for all the transcribed genes detected in the experiment.**
(XLS)

Table S2 **Modulation of phytoalexin genes reported as DESeq-normalized read counts in response to infection (24 h ) in GV** ***vs*** **VN rice genotypes.**
(DOC)

Table S3 **List of the WRKY genes (as determined by INTERPRO id: IPR003657; “DNA-binding WRKY”) expression values reported as DESeq-normalized read counts detected in GV and VN.**
(XLS)

Table S4 **Predicted WRKY isoforms detected for the rice genotypes GV and VN rice genotypes.**
(DOC)

Table S5 **Expression values reported as DESeq-normalized read counts of selected genes containing the INTERPRO LYS-M domain (IPR018392; Peptidoglycan-binding lysin domain) in the two GV and VN rice genotypes.**
(DOC)

Table S6 **Comparison of GV and VN expression values reported as DESeq-normalized read counts for MAPK, MAPKK and MAPKKK in GV and VN rice genotypes.**
(DOC)

Table S7 **Predicted MAP3K isoforms detected for GV and VN rice genotypes.**
(DOC)

Table S8 **Expression values, as DESeq normalized expression values, for GV and VN genes tagged by the INTERPRO domain IPR000767 (“disease resistance protein”).**
(XLS)

Table S9 **Resistance gene analogs (RGAs) candidates for the GV resistance.**
(DOC)

Table S10 **List of the GV DEG used in the Pearson co-regulation analysis.** For each gene, the name of the Affymetrix probe, the LOC name according to MSU Rice Genome Annotation Project, putative function (annotation) and gene symbol adopted in Figure 11 are reported. The Pearson correlation matrix based upon logarithmic values used to drawn Figure 11 is also included.
(XLS)

Table S11 **List of the gene loci whose transcription profile was evaluated by qRT-PCR.** For each gene the forward (fw) and reverse (rev) primer sequences are provided.
(DOC)
