# Supplementary figures and images for: Comparative Transcriptome Profiling of the Early Response to Magnaporthe oryzae in Durable Resistant vs Susceptible Rice (Oryza sativa L.) Genotypes
Source: PLoS One. 2012 Dec 12;7(12):e51609. doi: 10.1371/journal.pone.0051609 (PMC3520944; doi:10.1371/journal.pone.0051609)

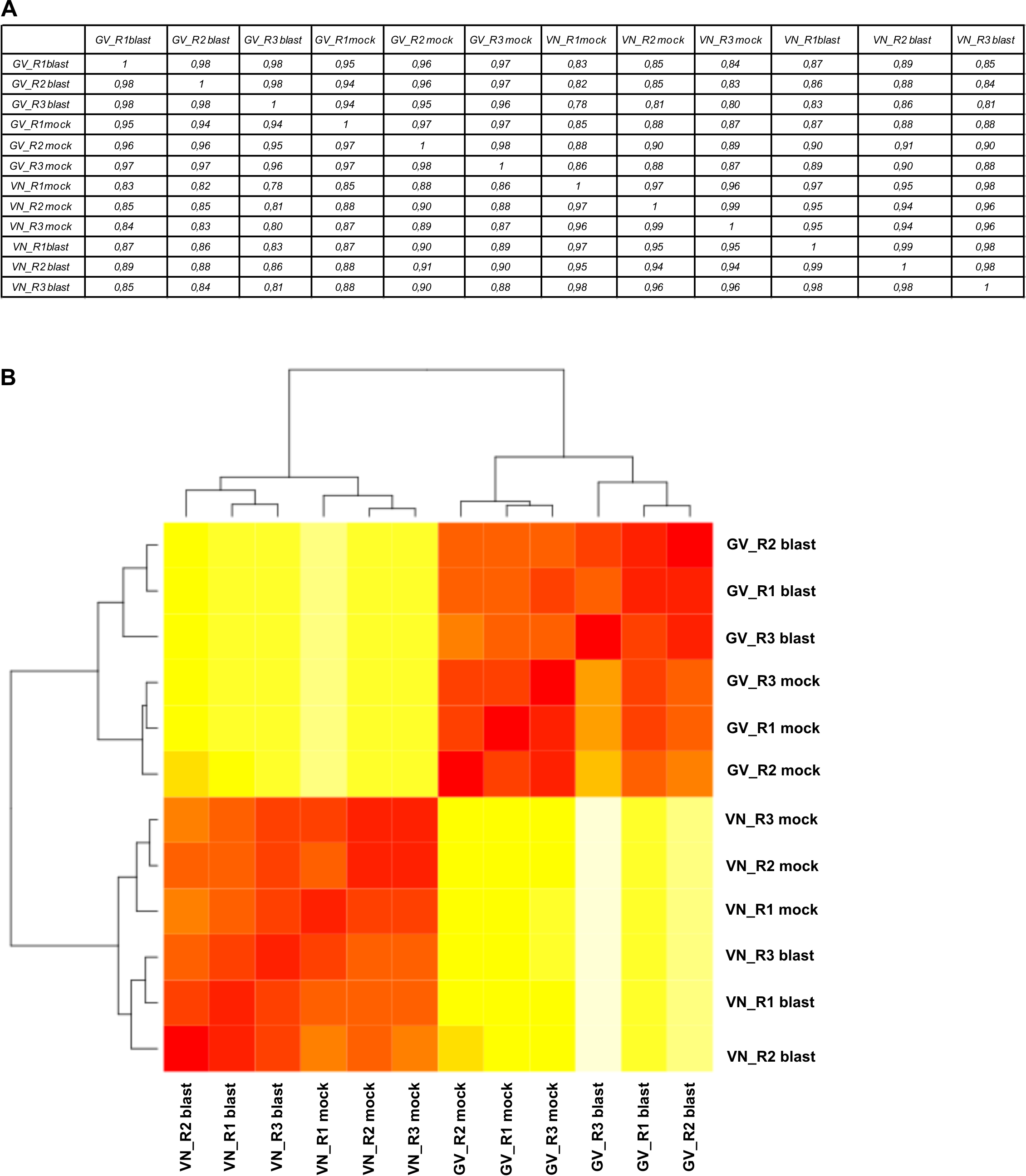

Supplement: Figure S1 — Spearman correlations among GV and VN samples. Panel A: Pairwise Spearman correlation (a non-parametric measure of statistical dependence between two variables) coefficients of the expression values in the different GV and VN treatments (blast or mock inoculated) and biological replicates (R1, R2, R3). Panel B: heatmap of the Spearman correlation coefficient for the expression values in the different GV and VN treatments (blast or mock inoculated) and biological replicates (R1, R2, R3). The color scale indicates the degree of correlation (white-yellow, low correlation; orange-red, strong correlation). (TIF) [file pone.0051609.s001.tif]

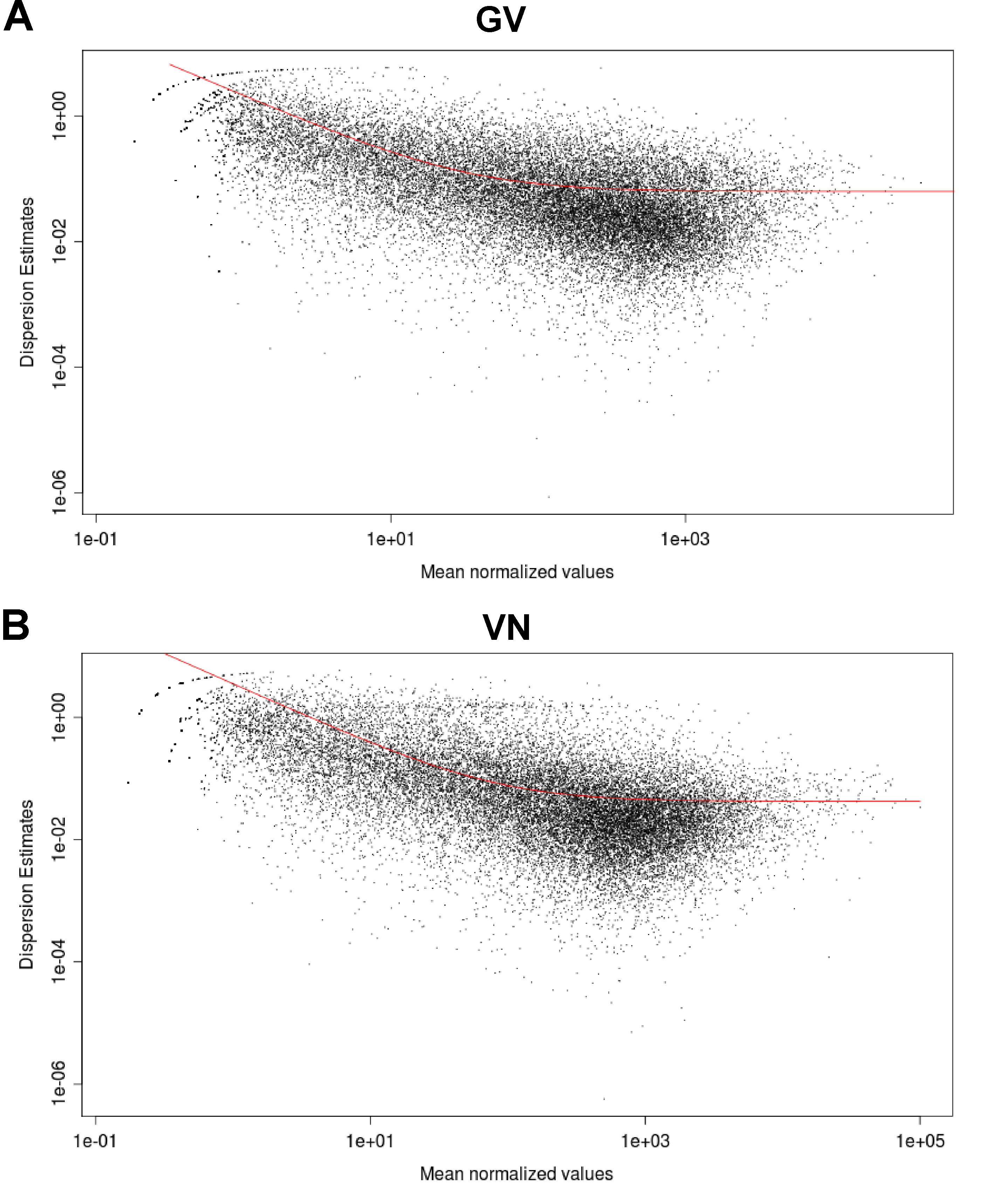

Supplement: Figure S2 — DESeq dispersion values plotted against means for GV (A) and VN (B). Empirical (black dots) and fitted (red lines) values are shown. (TIF) [file pone.0051609.s002.tif]
