# Supplementary material for: Comparative Transcriptome Profiling of the Early Response to Magnaporthe oryzae in Durable Resistant vs Susceptible Rice (Oryza sativa L.) Genotypes
Source: PLoS One. 2012 Dec 12;7(12):e51609. doi: 10.1371/journal.pone.0051609 (PMC3520944; doi:10.1371/journal.pone.0051609)
Supplement: Table S2 — Modulation of phytoalexin genes reported as DESeq-normalized read counts in response to infection (24 h ) in GV vs VN rice genotypes. (DOC) [file pone.0051609.s004.doc]

**Table S2.** Modulation of phytoalexin genes reported as DESeq-normalized read counts in response to infection (24 h ) in GV vs VN rice genotypes.

| ID MSU | Gene name (synonims) | Description MSU  (Description RAP) | variety | mock | blast | Fold induction | FDR | DEG |
| --- | --- | --- | --- | --- | --- | --- | --- | --- |
| LOC_Os02g36210 | CPS2  (OsCyc2) | ent-kaurene synthase, chloroplast precursor, putative, expressed | GV | 12.43 | 128.54 | 10.34 | 4.034E-04 | YES |
|  |  |  | VN | 1.68 | 17.13 | 10.18 | 0.2840 | NO |
| LOC_Os04g09900 | CPS4  (OsCyc1)  (OsCPS4) | ent-kaurene synthase, chloroplast precursor, putative, expressed | GV | 70.93 | 515.35 | 7.27 | 0.0034 | YES |
|  |  |  | VN | 8.87 | 100.47 | 11.32 | 0.2630 | NO |
| LOC_Os04g10060 | KS4,  OsKSL4 | ent-kaurene synthase, chloroplast precursor, putative, expressed | GV | 3.97 | 40.69 | 10.25 | 0.0001 | YES |
|  |  |  | VN | 1.98 | 3.98 | 2.01 | 0.9870 | NO |
| LOC_Os02g36140 | KS7  (OsKSL7) | terpene synthase, putative, expressed | GV | 25.02 | 358.03 | 14.31 | 5.123E-14 | YES |
|  |  |  | VN | 7.17 | 53.59 | 7.47 | 0.2790 | NO |
| LOC_Os11g28530 | KS8  (OsKSL8) | terpene synthase, putative, expressed  (Similar to Ent-kaurene synthase 1A) | GV | 86.12 | 388.47 | 4.51 | 0.0008 | YES |
|  |  |  | VN | 11.17 | 74.96 | 6.71 | 0.3960 | NO |
| LOC_Os04g10000 | OsMASL | sex determination protein tasselseed-2, putative, expressed | GV | 12.37 | 109.29 | 8.84 | 0.0001 | YES |
|  |  |  | VN | 3.91 | 14.56 | 3.72 | 0.2800 | NO |
| LOC_Os04g10010 | MAS1  (OsMAS) | sex determination protein tasselseed-2, putative, expressed | GV | 15.72 | 273.12 | 17.38 | 1.904E-05 | YES |
|  |  |  | VN | 2.68 | 8.23 | 3.07 | 0.75 | NO |
| LOC_Os04g10160 | CYP99A2 | cytochrome P450, putative, expressed | GV | 60.53 | 310.33 | 5.13 | 0.0033 | YES |
|  |  |  | VN | 8.00 | 37.42 | 4.68 | 0.1884 | NO |
