# Supplementary material for: Comparative Transcriptome Profiling of the Early Response to Magnaporthe oryzae in Durable Resistant vs Susceptible Rice (Oryza sativa L.) Genotypes
Source: PLoS One. 2012 Dec 12;7(12):e51609. doi: 10.1371/journal.pone.0051609 (PMC3520944; doi:10.1371/journal.pone.0051609)
Supplement: Table S4 — Predicted WRKY isoforms detected for the rice genotypes GV and VN rice genotypes. (DOC) [file pone.0051609.s006.doc]

**Table S4. Predicted WRKY isoforms for GV and VN rice genotypes. Class code “J”: Potentially novel isoform. Class code “=” : Complete match of intron chain with reference; OK : an outcome for test was reached; NOTEST: too few alignments were available for testing; LOWDATA: too complex or shallowly sequenced. Significant call “NO” includes no outcome tests.**

| **GV** | | | | | | | | | | | | | |
| --- | --- | --- | --- | --- | --- | --- | --- | --- | --- | --- | --- | --- | --- |
| nearest_ref_id | | MSU locus Description | TSS group id | Class code | length | status | | FPKM mock | | FPKM blast | FDR | Fold ch | significant |
| LOC_Os01g14440.1 | | OsWRKY1v2 - Superfamily of TFs having WRKY and zinc finger domains, expressed | TSS762 | j | 2518 | OK | | 27.85 | | 69.05 | 0.010 | 2.48 | yes |
| LOC_Os01g14440.1 | | OsWRKY1v2 - Superfamily of TFs having WRKY and zinc finger domains, expressed | TSS763 | = | 2114 | OK | | 25.62 | | 66.12 | 0.010 | 2.58 | yes |
| LOC_Os03g20550.1 | | OsWRKY55 - Superfamily of TFs having WRKY and zinc finger domains, expressed | TSS29504 | = | 911 | OK | | 4 | | 9.72 | 0.160 | 2.43 | no |
| LOC_Os03g20550.2 | | OsWRKY55 - Superfamily of TFs having WRKY and zinc finger domains, expressed | TSS29506 | = | 785 | OK | | 3.65 | | 7.78 | 0.320 | 2.13 | no |
| LOC_Os03g20550.3 | | OsWRKY55 - Superfamily of TFs having WRKY and zinc finger domains, expressed | TSS29505 | = | 1095 | OK | | 0.18 | | 1.49 | 0.010 | 8.27 | yes |
| LOC_Os03g45450.1 | | OsWRKY60 - Superfamily of TFs having WRKY and zinc finger domains, expressed | TSS33907 | j | 1526 | OK | | 1.72 | | 5.60 | 0.040 | 3.26 | yes |
| LOC_Os03g45450.1 | | OsWRKY60 - Superfamily of TFs having WRKY and zinc finger domains, expressed | TSS33907 | = | 542 | OK | | 2.68 | | 8.57 | 0.080 | 3.20 | no |
| LOC_Os05g09020.1 | | OsWRKY67 - Superfamily of TFs having WRKY and zinc finger domains, expressed | TSS44439 | = | 890 | OK | | 32.79 | | 86.85 | 0.020 | 2.65 | yes |
| LOC_Os05g09020.2 | | OsWRKY67 - Superfamily of TFs having WRKY and zinc finger domains, expressed | TSS44439 | = | 901 | OK | | 1.08 | | 5.30 | 0.020 | 4.92 | yes |
| LOC_Os07g48260.1 | | OsWRKY47 - Superfamily of TFs having WRKY and zinc finger domains, expressed | TSS57374 | j | 3119 | OK | | 0.18 | | 0.53 | 0.220 | 2.98 | no |
| LOC_Os07g48260.1 | | OsWRKY47 - Superfamily of TFs having WRKY and zinc finger domains, expressed | TSS57374 | j | 2704 | OK | | 0.66 | | 5.64 | 5.919E-06 | 8.54 | yes |
| LOC_Os07g48260.1 | | OsWRKY47 - Superfamily of TFs having WRKY and zinc finger domains, expressed | TSS57374 | = | 2269 | OK | | 2.27 | | 24.03 | 7.094E-10 | 10.59 | yes |
| LOC_Os08g38990.1 | | OsWRKY30 - Superfamily of TFs having WRKY and zinc finger domains, expressed | TSS62023 | = | 2424 | OK | | 2.33 | | 1.21 | 0.400 | 0.52 | no |
| LOC_Os08g38990.3 | | OsWRKY30 - Superfamily of TFs having WRKY and zinc finger domains, expressed | TSS62022 | = | 2502 | OK | | 0.69 | | 0 | 0.020 | 0 | yes |
| LOC_Os08g38990.4 | | OsWRKY30 - Superfamily of TFs having WRKY and zinc finger domains, expressed | TSS62023 | = | 2659 | NOTEST | | 0 | | 0 | 1.000 | Inf | no |
| LOC_Os08g38990.4 | | OsWRKY30 - Superfamily of TFs having WRKY and zinc finger domains, expressed | TSS62023 | = | 2608 | LOWDATA | | 0.73 | | 0.60 | 1.000 | 0.81 | no |
| LOC_Os09g25070.1 | | OsWRKY62 - Superfamily of TFs having WRKY and zinc finger domains, expressed | TSS65605 | = | 1330 | OK | | 0.29 | | 37.34 | 0.000E+00 | 128.43 | yes |
| LOC_Os09g25070.2 | | OsWRKY62 - Superfamily of TFs having WRKY and zinc finger domains, expressed | TSS65605 | = | 1463 | OK | | 1.63 | | 45.95 | 0.000E+00 | 28.19 | yes |
| LOC_Os11g02530.1 | | OsWRKY40 - Superfamily of TFs having WRKY and zinc finger domains, expressed | TSS12268 | x | 2055 | OK | | 1.38 | | 1.32 | 0.990 | 0.96 | no |
| LOC_Os11g02530.1 | | OsWRKY40 - Superfamily of TFs having WRKY and zinc finger domains, expressed | TSS14634 | = | 3060 | OK | | 0.17 | | 1.18 | 2.786E-03 | 7.04 | yes |
| LOC_Os11g02530.1 | | OsWRKY40 - Superfamily of TFs having WRKY and zinc finger domains, expressed | TSS14635 | = | 1210 | LOWDATA | | 0.75 | | 30.7 | 1.000 | 41.19 | no |
| LOC_Os12g02470.1 | | OsWRKY65 - Superfamily of TFs having WRKY and zinc finger domains, expressed | TSS19475 | j | 1443 | OK | | 0 | | 1.39 | 0.010 | Inf | yes |
| LOC_Os12g02470.1 | | OsWRKY65 - Superfamily of TFs having WRKY and zinc finger domains, expressed | TSS19476 | = | 1026 | OK | | 0 | | 4.04 | 0.010 | Inf | yes |
| **VN** | | | | | | | | | | | | | |
| nearest_ref_id | MSU locus Description | | TSS group id | Class code | length | status | FPKM contr | | FPKM stress | | FDR | Fold ch | significant |
| LOC_Os01g18584.1 | OsWRKY9 - Superfamily of TFs having WRKY and zinc finger domains, expressed | | TSS980 | j | 1777 | OK | 2.61 | | 8.51 | | 0.020 | 3.26 | yes |
| LOC_Os01g18584.1 | OsWRKY9 - Superfamily of TFs having WRKY and zinc finger domains, expressed | | TSS980 | = | 1832 | OK | 2.78 | | 14.56 | | 1.553E-04 | 5.24 | yes |
| LOC_Os05g27730.1 | OsWRKY53 - Superfamily of TFs having WRKY and zinc finger domains, expressed | | TSS43549 | j | 2785 | OK | 1.2 | | 3.6 | | 0.060 | 2.99 | no |
| LOC_Os05g27730.1 | OsWRKY53 - Superfamily of TFs having WRKY and zinc finger domains, expressed | | TSS43550 | = | 2075 | OK | 51.64 | | 107.01 | | 0.030 | 2.07 | yes |
| LOC_Os08g38990.1 | OsWRKY30 - Superfamily of TFs having WRKY and zinc finger domains, expressed | | TSS63298 | = | 2424 | OK | 3.8 | | 6.2 | | 0.460 | 1.63 | no |
| LOC_Os08g38990.3 | OsWRKY30 - Superfamily of TFs having WRKY and zinc finger domains, expressed | | TSS63299 | = | 2502 | LOWDATA | 0.44 | | 0 | | 1.000 | 0 | no |
| LOC_Os08g38990.4 | OsWRKY30 - Superfamily of TFs having WRKY and zinc finger domains, expressed | | TSS63298 | j | 2698 | OK | 0.39 | | 0 | | 0.050 | 0 | yes |
| LOC_Os08g38990.4 | OsWRKY30 - Superfamily of TFs having WRKY and zinc finger domains, expressed | | TSS63298 | j | 3240 | OK | 1.68 | | 2.3 | | 0.750 | 1.37 | no |
| LOC_Os08g38990.4 | OsWRKY30 - Superfamily of TFs having WRKY and zinc finger domains, expressed | | TSS63298 | = | 2659 | OK | 0.49 | | 1.55 | | 0.110 | 3.18 | no |
| LOC_Os08g38990.4 | OsWRKY30 - Superfamily of TFs having WRKY and zinc finger domains, expressed | | TSS63300 | = | 2608 | LOWDATA | 1.22 | | 2.84 | | 1.000 | 2.32 | no |
| LOC_Os09g25070.1 | OsWRKY62 - Superfamily of TFs having WRKY and zinc finger domains, expressed | | TSS67008 | = | 1330 | OK | 0.18 | | 0.02 | | 0.370 | 0.11 | no |
| LOC_Os09g25070.2 | OsWRKY62 - Superfamily of TFs having WRKY and zinc finger domains, expressed | | TSS67008 | = | 1463 | OK | 0.15 | | 1.13 | | 0.010 | 7.31 | yes |
| LOC_Os11g02480.2 | OsWRKY46 - Superfamily of TFs having WRKY and zinc finger domains, expressed | | TSS14904 | = | 1074 | OK | 9.07 | | 4.85 | | 0.360 | 0.53 | no |
| LOC_Os11g02480.2 | OsWRKY46 - Superfamily of TFs having WRKY and zinc finger domains, expressed | | TSS14904 | j | 1111 | OK | 0 | | 1.28 | | 0.05 | Inf | yes |
| LOC_Os12g02420.1 | WRKY DNA-binding domain containing protein, expressed | | TSS19846 | = | 1086 | OK | 9.52 | | 3.92 | | 0.14 | 0.41 | no |
| LOC_Os12g02420.2 | WRKY DNA-binding domain containing protein, expressed | | TSS19846 | = | 1080 | OK | 0.49 | | 4.46 | | 1.529E-03 | 9.03 | yes |
