# Supplementary material for: Comparative Transcriptome Profiling of the Early Response to Magnaporthe oryzae in Durable Resistant vs Susceptible Rice (Oryza sativa L.) Genotypes
Source: PLoS One. 2012 Dec 12;7(12):e51609. doi: 10.1371/journal.pone.0051609 (PMC3520944; doi:10.1371/journal.pone.0051609)
Supplement: Table S5 — Expression values reported as DESeq-normalized read counts of selected genes containing the INTERPRO LYS-M domain (IPR018392; Peptidoglycan-binding lysin domain) in the two GV and VN rice genotypes. (DOC) [file pone.0051609.s007.doc]

**Table S5** Expression values reported as DESeq-normalized read counts of selected genes containing the INTERPRO LYS-M domain (IPR018392; Peptidoglycan-binding lysin domain) in the two GV and VN rice genotypes.

| **GV** | | | | | |
| --- | --- | --- | --- | --- | --- |
| id | description | **mock** | **blast** | **Fold change** | **FDR** |
| LOC_Os01g57400 | lysM domain containing protein, putative, expressed | 0.99 | 3.13 | 3.16 | 1 |
| LOC_Os03g01990 | lysM domain containing protein, putative, expressed | 242.57 | 281.41 | 1.16 | 1 |
| LOC_Os03g02685 | lysM domain containing protein, putative, expressed | 918.82 | 1116.32 | 1.22 | 1 |
| LOC_Os03g04110 | lysM domain-containing GPI-anchored protein precursor, putative, expressed | 1032.09 | 1830.44 | 1.77 | 0.50 |
| LOC_Os03g49250 | OsFBO16 - F-box and other domain containing protein, expressed | 675.26 | 608.09 | 0.90 | 1 |
| LOC_Os04g48380 | lysM domain containing protein, putative, expressed | 6.23 | 16.92 | 2.72 | 0.32 |
| LOC_Os06g10660 | lysM domain-containing GPI-anchored protein 1 precursor, putative, expressed | 380.88 | 393.55 | 1.03 | 1 |
| LOC_Os06g51360 | lysM domain containing protein, putative, expressed | 686.62 | 858.02 | 1.25 | 0.99 |
| LOC_Os09g27890 | lysM domain-containing GPI-anchored protein precursor, putative, expressed | 37.34 | 16.99 | 0.46 | 0.84 |
| LOC_Os10g34420 | lysM domain containing protein, putative, expressed | 240.72 | 249.52 | 1.04 | 1 |
| LOC_Os10g38040 | lysM domain containing protein, putative, expressed | 252.71 | 449.87 | 1.78 | 0.66 |
| LOC_Os11g34570 | lysM domain-containing GPI-anchored protein precursor, putative | 4.32 | 5.12 | 1.19 | 1 |
| **VN** | | | | | |
| id | description | **mock** | **blast** | **Fold change** | **FDR** |
| LOC_Os02g53000 | lysM domain-containing GPI-anchored protein precursor, putative, expressed | 27.55 | 45.56 | 1.65 | 0.58 |
| LOC_Os03g01990 | lysM domain containing protein, putative, expressed | 191.28 | 131.11 | 0.69 | 0.33 |
| LOC_Os03g02685 | lysM domain containing protein, putative, expressed | 425.09 | 494.27 | 1.16 | 0.90 |
| LOC_Os03g04110 | lysM domain-containing GPI-anchored protein precursor, putative, expressed | 614.69 | 1429.76 | 2.33 | 5.957E-04 |
| LOC_Os03g49250 | OsFBO16 - F-box and other domain containing protein, expressed | 936.97 | 896.41 | 0.96 | 1 |
| LOC_Os04g48380 | lysM domain containing protein, putative, expressed | 12.30 | 3.56 | 0.29 | 0.41 |
| LOC_Os06g10660 | lysM domain-containing GPI-anchored protein 1 precursor, putative, expressed | 370.26 | 580.19 | 1.57 | 0.15 |
| LOC_Os06g51360 | lysM domain containing protein, putative, expressed | 760.31 | 518.05 | 0.68 | 0.22 |
| LOC_Os09g27890 | lysM domain-containing GPI-anchored protein precursor, putative, expressed | 16.36 | 12.66 | 0.77 | 0.97 |
| LOC_Os10g34420 | lysM domain containing protein, putative, expressed | 159.56 | 173.75 | 1.09 | 1 |
| LOC_Os10g38040 | lysM domain containing protein, putative, expressed | 178.08 | 272.27 | 1.53 | 0.64 |
