# Supplementary material for: Comparative Transcriptome Profiling of the Early Response to Magnaporthe oryzae in Durable Resistant vs Susceptible Rice (Oryza sativa L.) Genotypes
Source: PLoS One. 2012 Dec 12;7(12):e51609. doi: 10.1371/journal.pone.0051609 (PMC3520944; doi:10.1371/journal.pone.0051609)
Supplement: Table S6 — Comparison of GV and VN expression values reported as DESeq-normalized read counts for MAPK, MAPKK and MAPKKK in GV and VN rice genotypes. (DOC) [file pone.0051609.s008.doc]

**Table S6** Comparison of Gigante Vercelli (GV) and Vialone Nano (VN) expression values reported as DESeq-normalized read counts for MAPK, MAPKK and MAPKKK.

| **MAP kinases** | | | | | | | | |
| --- | --- | --- | --- | --- | --- | --- | --- | --- |
| id | Gene name | description |  | Mean mocka | Mean blasta | Fold Ch blast vs mock | FDR | Listed among DEG |
| LOC_Os01g10430 |  | protein kinase family protein, putative, expressed | GV | 287.20 | 209.42 | 0.73 | 0.81 | NO |
|  |  |  | VN | 985.03 | 738.15 | 0.75 | 0.45 | NO |
| LOC_Os01g43910 |  | CGMC_MAPKCMGC_2.4 - CGMC includes CDA, MAPK, GSK3, and CLKC kinases, expressed | GV | 542.55 | 720.97 | 1.33 | 0.90 | NO |
|  |  |  | VN | 338.13 | 333.96 | 0.99 | 1 | NO |
| LOC_Os01g45620 | OsMPK21-2 | CGMC_MAPKCMGC_2.5 - CGMC includes CDA, MAPK, GSK3, and CLKC kinases, expressed | GV | 50.86 | 35.96 | 0.71 | 0.88 | NO |
|  |  |  | VN | 217.40 | 141.00 | 0.65 | 0.30 | NO |
| LOC_Os01g47530 | OsMPK20-4 | CGMC_MAPKCMGC_2.6 - CGMC includes CDA, MAPK, GSK3, and CLKC kinases, expressed | GV | 1367.28 | 946.18 | 0.69 | 0.93 | NO |
|  |  |  | VN | 2927.55 | 2879.22 | 0.98 | 1 | NO |
| LOC_Os01g70130 |  | protein kinase domain containing protein, expressed | GV | 489.92 | 951.29 | 1.94 | 0.14 | NO |
|  |  |  | VN | 354.11 | 506.70 | 1.43 | 0.60 | NO |
| LOC_Os02g04230 | OsMPK17-2 | CGMC_MAPKCMGC_2.7 - CGMC includes CDA, MAPK, GSK3, and CLKC kinases, expressed | GV | 232.75 | 487.21 | 2.09 | 0.06 | NO |
|  |  |  | VN | 380.70 | 383.71 | 1.01 | 1 | NO |
| LOC_Os02g05480 | OsMPK14 | CGMC_MAPKCMGC_2_SLT2y_ERK.1 - CGMC includes CDA, MAPK, GSK3, and CLKC kinases, expressed | GV | 1481.07 | 1494.76 | 1.01 | 1 | NO |
|  |  |  | VN | 1529.20 | 1576.47 | 1.03 | 1 | NO |
| LOC_Os03g17700 | OsMPK3 | CGMC_MAPKCGMC_2_ERK.2 - CGMC includes CDA, MAPK, GSK3, and CLKC kinases, expressed | GV | 738.53 | 1737.56 | 2.35 | 0.01 | YES |
|  |  |  | VN | 167.94 | 339.86 | 2.02 | 0.08 | NO |
| LOC_Os03g18210 |  | basic helix-loop-helix, putative, expressed | GV | 84.53 | 87.11 | 1.03 | 1 | NO |
|  |  |  | VN | 226.94 | 216.55 | 0.95 | 1 | NO |
| LOC_Os03g22700 |  | cyclin-dependent kinase C-2, putative, expressed | GV | 57.02 | 61.07 | 1.07 | 1 | NO |
|  |  |  | VN | 160.01 | 197.05 | 1.23 | 0.79 | NO |
| LOC_Os04g41100 |  | cyclin-dependent kinase G-2, putative, expressed | GV | 1724.88 | 1669.89 | 0.97 | 1 | NO |
|  |  |  | VN | 2624.90 | 3249.83 | 1.24 | 0.71 | NO |
| LOC_Os05g01780 |  | STE_PAK_Ste20++TranslationKinase_Slob_Wnk.1 - STE kinases include homologs to sterile 7, sterile 11 and sterile 20 from yeast, expressed | GV | 171.93 | 199.22 | 1.16 | 1 | NO |
|  |  |  | VN | 183.18 | 167.11 | 0.91 | 1 | NO |
| LOC_Os05g05160 |  | CGMC_MAPKCGMC_2.1 - CGMC includes CDA, MAPK, GSK3, and CLKC kinases, expressed | GV | 2432.82 | 2706.93 | 1.11 | 1 | NO |
|  |  |  | VN | 2424.19 | 2129.93 | 0.88 | 0.84 | NO |
| LOC_Os05g49140 | OsMPK20-5 | CGMC_MAPKCMGC_2.8 - CGMC includes CDA, MAPK, GSK3, and CLKC kinases, expressed | GV | 868.39 | 715.59 | 0.82 | 1 | NO |
|  |  |  | VN | 1943.89 | 1112.11 | 0.57 | 0.03 | YES |
| LOC_Os05g50120 | OsMPK21-1 | CGMC_MAPKCMGC_2.3 - CGMC includes CDA, MAPK, GSK3, and CLKC kinases, expressed | GV | 29.82 | 23.34 | 0.78 | 1 | NO |
|  |  |  | VN | 72.14 | 76.02 | 1.05 | 1 | NO |
| LOC_Os06g06090 | OsMPK6 | CGMC_MAPKCMGC_2_ERK.12 - CGMC includes CDA, MAPK, GSK3, and CLKC kinases, expressed | GV | 840.71 | 1081.66 | 1.29 | 0.88 | NO |
|  |  |  | VN | 965.25 | 1013.23 | 1.05 | 1 | NO |
| LOC_Os06g26340 | OsMPK20-3 | CGMC_MAPKCMGC_2.10 - CGMC includes CDA, MAPK, GSK3, and CLKC kinases, expressed | GV | 538.91 | 463.03 | 0.86 | 1 | NO |
|  |  |  | VN | 2755.95 | 2284.51 | 0.83 | 0.66 | NO |
| LOC_Os06g48590 | OsMPK7 | CGMC_MAPKCMGC_2_SLT2y_ERK.2 - CGMC includes CDA, MAPK, GSK3, and CLKC kinases, expressed | GV | 1628.89 | 1945.04 | 1.19 | 1 | NO |
|  |  |  | VN | 1065.34 | 1903.98 | 1.79 | 0.03 | YES |
| LOC_Os06g49430 | OsMPK17-1 | CGMC_MAPKCMGC_2.11 - CGMC includes CDA, MAPK, GSK3, and CLKC kinases, expressed | GV | 2781.54 | 3389.77 | 1.22 | 1 | NO |
|  |  |  | VN | 2121.62 | 2261.10 | 1.07 | 1 | NO |
| LOC_Os07g47180 |  | transposon protein, putative, unclassified, expressed (putative CRK1 protein(cdc2-related kinase 1) [Oryza sativa Japonica Group) | GV | 477.61 | 338.03 | 0.71 | 0.69 | NO |
|  |  |  | VN | 590.82 | 608.53 | 1.03 | 1 | NO |
| LOC_Os08g06060 |  | CGMC_MAPKCMGC_2_ERK.13 - CGMC includes CDA, MAPK, GSK3, and CLKC kinases, expressed | GV | 76.01 | 56.14 | 0.74 | 0.88 | NO |
|  |  |  | VN | 167.90 | 139.85 | 0.83 | 0.87 | NO |
| LOC_Os08g35220 |  | cyclin-dependent kinase C-3, putative, expressed | GV | 66.05 | 53.11 | 0.80 | 1 | NO |
|  |  |  | VN | 106.65 | 107.58 | 1.01 | 1 | NO |
| LOC_Os10g38950 | OsMPK4 | CGMC_MAPKCMGC_2_ERK.14 - CGMC includes CDA, MAPK, GSK3, and CLKC kinases, expressed | GV | 763.64 | 1194.73 | 1.56 | 0.48 | NO |
|  |  |  | VN | 870.98 | 1037.14 | 1.19 | 0.84 | NO |
| LOC_Os10g42950 |  | cyclin-dependent kinase E-1, putative, expressed | GV | 1054.75 | 1074.73 | 1.02 | 1 | NO |
|  |  |  | VN | 1598.69 | 1687.03 | 1.06 | 1 | NO |
| LOC_Os11g13860 |  | cyclin-dependent kinase, putative, expressed | GV | 192.10 | 110.5 | 0.58 | 0.36 | NO |
|  |  |  | VN | 144.48 | 98.34 | 0.68 | 0.45 | NO |
| LOC_Os11g17080 | OsMPK16 | OsMPK15 - Putative MAPK based on amino acid sequence homology, expressed | GV | 84.48 | 77.23 | 0.91 | 1 | NO |
|  |  |  | VN | 176.60 | 151.83 | 0.86 | 0.89 | NO |
| LOC_Os12g06490 |  | STE_PAK_Ste20_Slob_Wnk.6 - STE kinases include homologs to sterile 7, sterile 11 and sterile 20 from yeast, expressed | GV | 535.15 | 546.73 | 1.02 | 1 | NO |
|  |  |  | VN | 1415.48 | 1148.67 | 0.81 | 0.70 | NO |
| LOC_Os12g42020 |  | AGC_PVPK_like_kin82y.20 - ACG kinases include homologs to PKA, PKG and PKC, expressed | GV | 0 | 2.21 | Inf | 0.87 | NO |
|  |  |  | VN | - | - | - | - | ND |

| **MAPKK** | | | | | | | | |
| --- | --- | --- | --- | --- | --- | --- | --- | --- |
| id | Gene name | description |  | Mean mock | Mean blast | Fold Ch blast vs mock | FDR | DEG |
| LOC_Os06g05520 | OsMKK1 | OsMKK1 - putative MAPKK based on amino acid sequence homology, expressed | GV | 362.12 | 612.92 | 1.69 | 0.27 | NO |
|  |  |  | VN | 752.38 | 756.10 | 1.01 | 1 | NO |
| LOC_Os06g27890 | OsMKK3 | STE_MEK_ste7_MAP2K.8 - STE kinases include homologs to sterile 7, sterile 11 and sterile 20 from yeast, expressed | GV | 257.96 | 306.73 | 1.19 | 0.54 | NO |
|  |  |  | VN | 746.21 | 575.39 | 0.77 | 0.56 | NO |
| LOC_Os02g54600 | OsMKK4 | STE_MEK_ste7_MAP2K.5 - STE kinases include homologs to sterile 7, sterile 11 and sterile 20 from yeast, expressed | GV | 881.41 | 1369.15 | 1.55 | 0.51 | NO |
|  |  |  | VN | 268.33 | 567.69 | 2.12 | 0.03 | YES |
| LOC_Os06g09180 | OsMKK5 | OsMKK5 - putative MAPKK based on amino acid sequence homology, expressed | GV | 870.32 | 802.64 | 0.92 | 1 | NO |
|  |  |  | VN | 619.40 | 927.7 | 1.50 | 0.21 | NO |
| LOC_Os01g32660 | OsMKK6 | STE_MEK_ste7_MAP2K.2 - STE kinases include homologs to sterile 7, sterile 11 and sterile 20 from yeast, expressed | GV | 751.75 | 1084.31 | 1.44 | 0.68 | NO |
|  |  |  | VN | 1684.04 | 1513.31 | 0.90 | 0.93 | NO |
| LOC_Os02g46760 | OsMKK10-1 | STE_MEK_ste7_MAP2K.4 - STE kinases include homologs to sterile 7, sterile 11 and sterile 20 from yeast | GV | 2.70 | 1.68 | 0.63 | 1 | NO |
|  |  |  | VN | 0.28 | 1.30 | 4.64 | 1 | NO |
| LOC_Os03g12390 | OsMKK10-2 | STE_MEK_ste7_MAP2K.6 - STE kinases include homologs to sterile 7, sterile 11 and sterile 20 from yeast, expressed | GV | 522.30 | 984.34 | 1.89 | 0.10 | NO |
|  |  |  | VN | 709.54 | 1045.24 | 1.47 | 0.53 | NO |
| LOC_Os03g50550 | OsMKK10-3 | STE_MEK_ste7_MAP2K.7 - STE kinases include homologs to sterile 7, sterile 11 and sterile 20 from yeast | GV | 0 | 1.45 | Inf | 1 | NO |
|  |  |  | VN | 0 | 0 | 0 | ND | ND |

| **MAPKKK** | | | | | | | |
| --- | --- | --- | --- | --- | --- | --- | --- |
| **id** | **description** |  | **mean mock** | **mean blast** | **Fold Ch blast vs mock** | **FDR** | **DEG** |
| LOC_Os01g50370 | STE_MEKK_ste11_MAP3K.4 - STE kinases include homologs to sterile 7, sterile 11 and sterile 20 from yeast, expressed | GV | 221.44 | 376.15 | 1.70 | 0.97 | NO |
|  |  | VN | 41.45 | 30.74 | 0.74 | 0.86 | NO |
| LOC_Os01g50400 | STE_MEKK_ste11_MAP3K.5 - STE kinases include homologs to sterile 7, sterile 11 and sterile 20 from yeast, expressed | GV | 76.78 | 140.82 | 1.83 | 1 | NO |
|  |  | VN | 9.10 | 14.61 | 1.61 | 0.85 | NO |
| LOC_Os01g50410 | STE_MEKK_ste11_MAP3K.6 - STE kinases include homologs to sterile 7, sterile 11 and sterile 20 from yeast, expressed | GV | 177.42 | 381.99 | 2.15 | 0.56 | NO |
|  |  | VN | 11.31 | 19.19 | 1.70 | 0.68 | NO |
| LOC_Os01g50420 | STE_MEKK_ste11_MAP3K.7 - STE kinases include homologs to sterile 7, sterile 11 and sterile 20 from yeast, expressed | GV | 108.34 | 313.87 | 2.90 | 0.10 | NO |
|  |  | VN | ND | ND | ND | ND | ND |
| LOC_Os02g21700 | STE_MEKK_ste11_MAP3K.8 - STE kinases include homologs to sterile 7, sterile 11 and sterile 20 from yeast, expressed | GV | 323.42 | 556.61 | 1.72 | 0.62 | NO |
|  |  | VN | 46.82 | 32.77 | 0.7 | 0.93 | NO |
| LOC_Os02g35010 | STE_MEKK_ste11_MAP3K.9 - STE kinases include homologs to sterile 7, sterile 11 and sterile 20 from yeast, expressed | GV | 577.11 | 804.13 | 1.39 | 0.55 | NO |
|  |  | VN | 579.58 | 453.21 | 0.78 | 0.54 | NO |
| LOC_Os02g44642 | STE_MEKK_ste11_MAP3K.10 - STE kinases include homologs to sterile 7, sterile 11 and sterile 20 from yeast, expressed | GV | 417.62 | 334.12 | 0.8 | 0.97 | NO |
|  |  | VN | 937.28 | 639.25 | 0.68 | 0.23 | NO |
| LOC_Os02g53040 | STE_MEKK_ste11_MAP3K.11 - STE kinases include homologs to sterile 7, sterile 11 and sterile 20 from yeast, expressed | GV | 14.83 | 25.98 | 1.75 | 0.94 | NO |
|  |  | VN | 34.48 | 37.60 | 1.09 | 1 | NO |
| LOC_Os03g15570 | STE_MEKK_ste11_MAP3K.12 - STE kinases include homologs to sterile 7, sterile 11 and sterile 20 from yeast, expressed | GV | 674.99 | 995.12 | 1.47 | 0.65 | NO |
|  |  | VN | 862.87 | 797.86 | 0.93 | 0.98 | NO |
| LOC_Os03g18170 | STE_MEKK_ste11_MAP3K.13 - STE kinases include homologs to sterile 7, sterile 11 and sterile 20 from yeast, expressed | GV | 290.66 | 412.21 | 1.42 | 0.78 | NO |
|  |  | VN | 180.06 | 127.76 | 0.71 | 0.93 | NO |
| LOC_Os03g49640 | STE_MEKK_ste11_MAP3K.14 - STE kinases include homologs to sterile 7, sterile 11 and sterile 20 from yeast, expressed | GV | 875.38 | 1118.85 | 1.28 | 0.96 | NO |
|  |  | VN | 973.49 | 1140.25 | 1.17 | 0.87 | NO |
| LOC_Os03g55560 | STE_MEKK_ste11_MAP3K.15 - STE kinases include homologs to sterile 7, sterile 11 and sterile 20 from yeast, expressed | GV | 756.56 | 1000.40 | 1.32 | 0.93 | NO |
|  |  | VN | 938.52 | 978.96 | 1.04 | 1 | NO |
| LOC_Os04g35700 | STE_MEKK_ste11_MAP3K.16 - STE kinases include homologs to sterile 7, sterile 11 and sterile 20 from yeast, expressed | GV | 901.95 | 1115.15 | 1.24 | 0.99 | NO |
|  |  | VN | 2483.01 | 2512.91 | 1.01 | 1 | NO |
| LOC_Os04g47240 | STE_MEKK_ste11_MAP3K.17 - STE kinases include homologs to sterile 7, sterile 11 and sterile 20 from yeast, expressed | GV | 1162.29 | 1003.35 | 0.86 | 1 | NO |
|  |  | VN | 2014.93 | 1684.73 | 0.84 | 0.69 | NO |
| LOC_Os04g56530 | STE_MEKK_ste11_MAP3K.1 - STE kinases include homologs to sterile 7, sterile 11 and sterile 20 from yeast, expressed | GV | 894.40 | 974.66 | 1.09 | 1 | NO |
|  |  | VN | 1531.44 | 1451.18 | 0.95 | 0.98 | NO |
| LOC_Os05g46750 | STE_MEKK_ste11_MAP3K.18 - STE kinases include homologs to sterile 7, sterile 11 and sterile 20 from yeast, expressed | GV | 0 | 12.28 | Inf | 0.00 | YES |
|  |  | VN | ND | ND | ND | ND | ND |
| LOC_Os05g46760 | STE_MEKK_ste11_MAP3K.19 - STE kinases include homologs to sterile 7, sterile 11 and sterile 20 from yeast, expressed | GV | 25.93 | 71.5 | 2.76 | 0.03 | YES |
|  |  | VN | 6.45 | 19.73 | 3.06 | 0.28 | NO |
| LOC_Os07g02780 | STE_MEKK_ste11_MAP3K.20 - STE kinases include homologs to sterile 7, sterile 11 and sterile 20 from yeast, expressed | GV | 153.38 | 108.68 | 0.71 | 0.74 | NO |
|  |  | VN | 195.88 | 184.55 | 0.94 | 0.97 | NO |
| LOC_Os11g10100 | STE_MEKK_ste11_MAP3K.3 - STE kinases include homologs to sterile 7, sterile 11 and sterile 20 from yeast, expressed | GV | 1381.25 | 1805.76 | 1.31 | 0.71 | NO |
|  |  | VN | 1653.91 | 2846.62 | 1.72 | 0.22 | NO |

aExpression values are reported as DESeq-normalized read counts
