# Supplementary material for: Comparative Transcriptome Profiling of the Early Response to Magnaporthe oryzae in Durable Resistant vs Susceptible Rice (Oryza sativa L.) Genotypes
Source: PLoS One. 2012 Dec 12;7(12):e51609. doi: 10.1371/journal.pone.0051609 (PMC3520944; doi:10.1371/journal.pone.0051609)
Supplement: Table S7 — Predicted MAP3K isoforms detected for GV and VN rice genotypes. (DOC) [file pone.0051609.s009.doc]

**Table S7** Predicted MAP3K isoforms detected for GV and VN rice genotypes.

Class code “J”: Potentially novel isoform. Class code “=” : Complete match of intron chain with reference; OK : an outcome for test was reached; NOTEST: too few alignments were available for testing; LOWDATA: too complex or shallowly sequenced. Significant call “NO” includes no outcome tests.

|  | | | | | | | | | | | | |
| --- | --- | --- | --- | --- | --- | --- | --- | --- | --- | --- | --- | --- |
| **GV** | | | | | | | | | | | | |
| **Isoform id** | **TSS group id** | | **Class code** | **Nearest ref id** | **locus** | **length** | **status** | **mock** | **blast** | **Fold ch** | **FDR** | **significant** |
| TCONS_00016381 | TSS12688 | | j | LOC_Os11g10100.1 | 11:5455942-5462873 | 4008 | OK | 0.20 | 0.90 | 8.54 | 0.03 | yes |
| TCONS_00016382 | TSS12688 | | = | LOC_Os11g10100.2 | 11:5455942-5462873 | 1792 | OK | 0.49 | 0.56 | 1.20 | 0.96 | no |
| TCONS_00016383 | TSS12688 | | = | LOC_Os11g10100.1 | 11:5455942-5462873 | 2235 | LOWDATA | 46.13 | 68.32 | 1.76 | 1 | no |
| TCONS_00016384 | TSS12689 | | = | LOC_Os11g10100.3 | 11:5455942-5462873 | 2860 | OK | 1.48 | 1.88 | 1.42 | 0.86 | no |
| TCONS_00031153 | TSS24617 | | j | LOC_Os02g53040.1 | 2:32454810-32458836 | 3021 | OK | 2.53 | 3.23 | 1.43 | 0.83 | no |
| TCONS_00031154 | TSS24618 | | = | LOC_Os02g53040.1 | 2:32454810-32458836 | 911 | LOWDATA | 0.86 | 2.78 | 5.40 | 1 | no |
| TCONS_00031155 | TSS24619 | | j | LOC_Os02g53040.1 | 2:32454810-32458836 | 2643 | OK | 0.55 | 0.82 | 1.76 | 0.76 | no |
| TCONS_00034828 | TSS27343 | | = | LOC_Os02g44642.1 | 2:27043018-27056126 | 3757 | OK | 8.12 | 6.91 | 0.79 | 0.88 | no |
| TCONS_00034829 | TSS27343 | | j | LOC_Os02g44642.1 | 2:27043018-27056126 | 4825 | OK | 0.82 | 0.74 | 0.86 | 0.96 | no |
| TCONS_00041856 | TSS32470 | | = | LOC_Os03g15570.1 | 3:8571802-8576438 | 2660 | OK | 21.31 | 32.74 | 1.86 | 0.40 | no |
| TCONS_00044035 | TSS34149 | | = | LOC_Os03g49640.1 | 3:28262571-28268763 | 2537 | OK | 23.61 | 30.98 | 1.48 | 0.70 | no |
| TCONS_00044036 | TSS34149 | | = | LOC_Os03g49640.2 | 3:28262571-28268763 | 2631 | OK | 4.91 | 7.32 | 1.78 | 0.60 | no |
| TCONS_00044440 | TSS34432 | | j | LOC_Os03g55560.2 | 3:31605166-31612460 | 3392 | OK | 0.53 | 0.79 | 1.76 | 0.76 | no |
| TCONS_00044441 | TSS34432 | | = | LOC_Os03g55560.1 | 3:31605166-31612460 | 2999 | OK | 17.79 | 24.57 | 1.59 | 0.61 | no |
| TCONS_00044442 | TSS34432 | | = | LOC_Os03g55560.2 | 3:31605166-31612460 | 2918 | OK | 2.88 | 3.73 | 1.45 | 0.82 | no |
| TCONS_00051112 | TSS39866 | | j | LOC_Os04g35700.1 | 4:21578691-21586712 | 2609 | OK | 0.44 | 1.57 | 6.29 | 0.06 | no |
| TCONS_00051113 | TSS39866 | | = | LOC_Os04g35700.1 | 4:21578691-21586712 | 2743 | OK | 23.33 | 30.00 | 1.44 | 0.73 | no |
| TCONS_00051114 | TSS39866 | | = | LOC_Os04g35700.2 | 4:21578691-21586712 | 2046 | NOTEST | 0.11 | 0.17 | 2.01 | 1 | no |
| TCONS_00052015 | TSS40498 | | j | LOC_Os04g47240.1 | 4:27861401-27871972 | 6231 | OK | 1.07 | 1.47 | 1.58 | 0.76 | no |
| TCONS_00052016 | TSS40498 | | j | LOC_Os04g47240.1 | 4:27861401-27871972 | 6115 | OK | 8.96 | 10.63 | 1.28 | 0.85 | no |
| TCONS_00052017 | TSS40499 | | = | LOC_Os04g47240.2 | 4:27861401-27871972 | 4343 | OK | 1.00 | 0.79 | 0.71 | 0.87 | no |
| TCONS_00052018 | TSS40499 | | = | LOC_Os04g47240.3 | 4:27861401-27871972 | 4638 | OK | 0.47 | 1.05 | 3.18 | 0.28 | no |
| TCONS_00052019 | TSS40498 | | = | LOC_Os04g47240.1 | 4:27861401-27871972 | 4042 | OK | 11.41 | 7.03 | 0.50 | 0.38 | no |
| TCONS_00052759 | TSS41045 | | j | LOC_Os04g56530.1 | 4:33522308-33534170 | 4706 | OK | 12.57 | 6.97 | 0.43 | 0.21 | no |
| TCONS_00052760 | TSS41045 | | = | LOC_Os04g56530.1 | 4:33522308-33534170 | 4715 | OK | 3.18 | 10.96 | 5.95 | 2.054E-03 | yes |
| TCONS_00052761 | TSS41045 | | j | LOC_Os04g56530.1 | 4:33522308-33534170 | 860 | OK | 0.54 | 1.11 | 2.83 | 0.48 | no |
| TCONS_00059551 | TSS46352 | | = | LOC_Os05g46750.1 | 5:27004720-27007371 | 1842 | OK | 0 | 0.64 | Inf | 0.02 | yes |
| TCONS_00059552 | TSS46353 | | = | LOC_Os05g46760.1 | 5:27013302-27014721 | 1419 | OK | 1.62 | 4.78 | 4.78 | 0.06 | no |
| TCONS_00070461 | TSS55077 | | = | LOC_Os07g02780.1 | 7:1014337-1020609 | 2985 | OK | 3.53 | 2.5 | 0.61 | 0.71 | no |
| TCONS_00070462 | TSS55077 | | j | LOC_Os07g02780.1 | 7:1014337-1020609 | 2008 | OK | 1.07 | 1.04 | 0.96 | 0.99 | no |
| **VN** | | | | | | | | | | | | |
| **isoform_id** | | **TSS_group_id** | **class_code** | **nearest_ref_id** | **locus** | **length** | **status** | **mock** | **blast** | **fold_ch** | **FDR** | **significant** |
| TCONS_00003474 | | TSS2619 | = | LOC_Os01g50370.1 | 1:28920452-28922040 | 1588 | OK | 1.22 | 0.88 | 0.63 | 0.82 | no |
| TCONS_00003476 | | TSS2621 | = | LOC_Os01g50400.1 | 1:28932799-28934165 | 1366 | OK | 0.31 | 0.53 | 2.19 | 0.67 | no |
| TCONS_00008820 | | TSS6588 | = | LOC_Os01g50410.1 | 1:28937925-28939882 | 1356 | OK | 0.33 | 0.63 | 2.52 | 0.57 | no |
| TCONS_00008821 | | TSS6589 | = | LOC_Os01g50420.1 | 1:28944182-28947000 | 1646 | NOTEST | 0.07 | 0.08 | 1.09 | 1 | no |
| TCONS_00017043 | | TSS12919 | = | LOC_Os11g10100.2 | 11:5455951-5462137 | 1792 | LOWDATA | 0.17 | 0.64 | 6.64 | 1 | no |
| TCONS_00017044 | | TSS12919 | = | LOC_Os11g10100.1 | 11:5455951-5462137 | 2235 | OK | 30.65 | 51.91 | 2.14 | 0.19 | no |
| TCONS_00017045 | | TSS12919 | j | LOC_Os11g10100.1 | 11:5455951-5462137 | 4175 | OK | 0.09 | 0.23 | 3.95 | 0.38 | no |
| TCONS_00017046 | | TSS12919 | j | LOC_Os11g10100.1 | 11:5455951-5462137 | 4294 | OK | 0.21 | 0.15 | 0.64 | 0.86 | no |
| TCONS_00017047 | | TSS12919 | j | LOC_Os11g10100.1 | 11:5455951-5462137 | 5264 | NOTEST | 0.03 | 0.01 | 0.16 | 1 | no |
| TCONS_00017048 | | TSS12920 | = | LOC_Os11g10100.3 | 11:5455951-5462137 | 2860 | OK | 2.35 | 1.89 | 0.73 | 0.85 | no |
| TCONS_00030158 | | TSS23372 | = | LOC_Os02g21700.1 | 2:12893453-12895429 | 1416 | OK | 1.17 | 0.78 | 0.56 | 0.76 | no |
| TCONS_00032514 | | TSS25101 | j | LOC_Os02g53040.1 | 2:32452696-32458842 | 4388 | OK | 2.07 | 2.87 | 1.61 | 0.70 | no |
| TCONS_00032515 | | TSS25102 | j | LOC_Os02g53040.1 | 2:32452696-32458842 | 3105 | OK | 0.34 | 0.22 | 0.54 | 0.78 | no |
| TCONS_00032516 | | TSS25103 | = | LOC_Os02g53040.1 | 2:32452696-32458842 | 911 | LOWDATA | 1.18 | 1.53 | 1.46 | 1 | no |
| TCONS_00032517 | | TSS25103 | j | LOC_Os02g53040.1 | 2:32452696-32458842 | 3288 | NOTEST | 0.11 | 0 | 0 | 1 | no |
| TCONS_00035511 | | TSS27298 | = | LOC_Os02g35010.1 | 2:20996260-21002541 | 2636 | OK | 9.87 | 7.43 | 0.67 | 0.70 | no |
| TCONS_00036315 | | TSS27890 | = | LOC_Os02g44642.1 | 2:27043018-27056086 | 3757 | OK | 11.19 | 7.50 | 0.56 | 0.47 | no |
| TCONS_00039202 | | TSS29940 | = | LOC_Os03g18170.1 | 3:10183497-10189247 | 1536 | OK | 4.43 | 3.15 | 0.61 | 0.72 | no |
| TCONS_00043665 | | TSS33092 | = | LOC_Os03g15570.1 | 3:8571802-8576438 | 2660 | OK | 14.68 | 13.34 | 0.87 | 0.93 | no |
| TCONS_00045934 | | TSS34813 | = | LOC_Os03g49640.1 | 3:28262571-28268763 | 2537 | OK | 15.67 | 18.87 | 1.31 | 0.82 | no |
| TCONS_00045935 | | TSS34813 | = | LOC_Os03g49640.2 | 3:28262571-28268763 | 2631 | OK | 1.91 | 1.38 | 0.63 | 0.78 | no |
| TCONS_00046353 | | TSS35101 | = | LOC_Os03g55560.1 | 3:31605281-31612424 | 2999 | OK | 10.14 | 11.17 | 1.15 | 0.93 | no |
| TCONS_00046354 | | TSS35101 | = | LOC_Os03g55560.2 | 3:31605281-31612424 | 2918 | OK | 4.40 | 3.65 | 0.76 | 0.86 | no |
| TCONS_00053347 | | TSS40662 | = | LOC_Os04g35700.1 | 4:21580609-21586726 | 2743 | OK | 32.77 | 32.68 | 1.00 | 1.00 | no |
| TCONS_00053348 | | TSS40662 | j | LOC_Os04g35700.1 | 4:21580609-21586726 | 2753 | OK | 6.94 | 6.63 | 0.94 | 0.97 | no |
| TCONS_00053349 | | TSS40662 | j | LOC_Os04g35700.1 | 4:21580609-21586726 | 3413 | OK | 2.14 | 1.44 | 0.56 | 0.66 | no |
| TCONS_00053350 | | TSS40662 | = | LOC_Os04g35700.2 | 4:21580609-21586726 | 2046 | NOTEST | 0.05 | 0 | 0 | 1 | no |
| TCONS_00054246 | | TSS41291 | = | LOC_Os04g47240.2 | 4:27864106-27871972 | 4343 | OK | 2.82 | 0.99 | 0.22 | 0.06 | no |
| TCONS_00054247 | | TSS41291 | = | LOC_Os04g47240.3 | 4:27864106-27871972 | 4638 | OK | 0.64 | 0.76 | 1.30 | 0.91 | no |
| TCONS_00054248 | | TSS41292 | j | LOC_Os04g47240.1 | 4:27864106-27871972 | 4037 | NOTEST | 0.00 | 0 | 0 | 1 | no |
| TCONS_00054249 | | TSS41292 | = | LOC_Os04g47240.1 | 4:27864106-27871972 | 4042 | OK | 19.20 | 16.38 | 0.80 | 0.84 | no |
| TCONS_00054999 | | TSS41842 | = | LOC_Os04g56530.1 | 4:33519765-33534138 | 6483 | NOTEST | 0 | 0 | 1 | 1 | no |
| TCONS_00055000 | | TSS41842 | j | LOC_Os04g56530.1 | 4:33519765-33534138 | 6474 | OK | 1.07 | 0.82 | 0.68 | 0.81 | no |
| TCONS_00055001 | | TSS41842 | = | LOC_Os04g56530.1 | 4:33519765-33534138 | 4715 | LOWDATA | 14.43 | 13.48 | 0.91 | 1 | no |
| TCONS_00062097 | | TSS47261 | = | LOC_Os05g46750.1 | 5:27004720-27007371 | 1842 | NOTEST | 0.02 | 0 | 0 | 1 | no |
| TCONS_00062098 | | TSS47262 | = | LOC_Os05g46760.1 | 5:27013302-27014721 | 1419 | OK | 0.23 | 0.70 | 5.05 | 0.18 | no |
| TCONS_00073515 | | TSS56165 | j | LOC_Os07g02780.1 | 7:1014337-1020624 | 3404 | OK | 0.39 | 0.29 | 0.67 | 0.87 | no |
| TCONS_00073516 | | TSS56165 | = | LOC_Os07g02780.1 | 7:1014337-1020624 | 2985 | OK | 2.09 | 2.05 | 0.97 | 0.99 | no |
| TCONS_00073517 | | TSS56165 | j | LOC_Os07g02780.1 | 7:1014337-1020624 | 3789 | OK | 0.49 | 0.33 | 0.55 | 0.77 | no |
|  | |  |  |  |  |  |  |  |  |  |  |  |
