# Supplementary material for: Comparative Transcriptome Profiling of the Early Response to Magnaporthe oryzae in Durable Resistant vs Susceptible Rice (Oryza sativa L.) Genotypes
Source: PLoS One. 2012 Dec 12;7(12):e51609. doi: 10.1371/journal.pone.0051609 (PMC3520944; doi:10.1371/journal.pone.0051609)
Supplement: Table S9 — Resistance gene analogs (RGAs) candidates for the GV resistance. (DOC) [file pone.0051609.s011.doc]

**Table S9. Resistance gene analogs (RGAs) candidates for the GV resistance**

| **id** | **MSU 6.12 description (alternative description)** | **GV** | | | | **VN** | | | |
| --- | --- | --- | --- | --- | --- | --- | --- | --- | --- |
|  |  | **GV mock** | **GV blast** | **fold ch GV blast vs GV mock** | **FDR GV** | **VN mock** | **VN blast** | **fold ch VN blast vs VN mock** | **FDR VN** |
| **RGAs significantly upregulated in GV only** | | | | | | | | | |
| LOC_Os09g34160 | resistance protein, putative, expressed | 17.99 | 421.59 | 23.43 | 2.420E-25 | 13.47 | 59.38 | 4.41 | 0.76 |
| LOC_Os02g39884 | expressed protein | 29.54 | 108.19 | 3.66 | 1.334E-03 | 24.87 | 43.45 | 1.75 | 0.72 |
| LOC_Os06g48520 | disease resistance protein RPM1, putative | 20.97 | 74.67 | 3.56 | 0.01 | 20.02 | 39.14 | 1.96 | 0.39 |
| LOC_Os04g02030 | rp1, putative | 33.70 | 134.98 | 4.01 | 0.01 | 29.36 | 38.76 | 1.32 | 0.93 |
| LOC_Os03g10910 | NB-ARC/LRR disease resistance protein, putative, expressed | 8.36 | 37.12 | 4.44 | 0.01 | 3.62 | 8.10 | 2.24 | 0.80 |
| LOC_Os08g07890 | NB-ARC domain containing protein | 21.28 | 361.11 | 16.97 | 0.02 | 32.66 | 137.75 | 4.22 | 0.18 |
| LOC_Os07g17250 | disease resistance RPP13-like protein 1, putative, expressed | 216.07 | 489.88 | 2.27 | 0.02 | 565.97 | 913.59 | 1.61 | 0.13 |
| LOC_Os09g34150 | NBS-LRR disease resistance protein, putative, expressed | 111.95 | 235.34 | 2.10 | 0.04 | 253.64 | 333.12 | 1.31 | 0.65 |
| **RGAs expressed in GV and not expressed in VN** | | | | | | | | | |
| LOC_Os01g16400 | NB-ARC domain containing protein, expressed | 2.54 | 4.61 | 1.81 | 1 | - | - | - | - |
| LOC_Os01g52320 | NB-ARC domain containing protein, expressed | 2.52 | 12.93 | 5.13 | 0.25 | - | - | - | - |
| LOC_Os01g57270 | disease resistance RPP13-like protein 1, putative, expressed | 32.25 | 48.13 | 1.49 | 0.86 | - | - | - | - |
| LOC_Os01g72700 | expressed protein | 8.08 | 12.17 | 1.51 | 1 | - | - | - | - |
| LOC_Os02g04530 | rust resistance-like protein RP1, putative | 7.99 | 43.13 | 5.40 | 0.28 | - | - | - | - |
| LOC_Os02g27540 | NB-ARC domain containing protein | 0 | 1.45 | Inf | 1 | - | - | - | - |
| LOC_Os03g63240 | disease resistance protein, putative, expressed | 2.96 | 0.84 | 0.29 | 1 | - | - | - | - |
| LOC_Os04g25900 | go35 NBS-LRR, putative, expressed | 12.56 | 28.62 | 2.28 | 0.37 | - | - | - | - |
| LOC_Os04g32940 | Leucine Rich Repeat family protein, expressed | 2.74 | 8.70 | 3.17 | 0.70 | - | - | - | - |
| LOC_Os04g53060 | NBS-LRR disease resistance protein, putative, expressed | 5.77 | 4.98 | 0.86 | 1 | - | - | - | - |
| LOC_Os05g12770 | NB-ARC domain containing protein | 0.90 | 3.88 | 4.32 | 0.96 | - | - | - | - |
| LOC_Os06g15750 | NB-ARC domain containing protein | 1.07 | 3.90 | 3.64 | 1 | - | - | - | - |
| LOC_Os07g27370 | resistance protein, putative, expressed | 1.07 | 3.13 | 2.92 | 1 | - | - | - | - |
| LOC_Os08g07340 | mla1, putative | 9.96 | 9.77 | 0.98 | 1 | - | - | - | - |
| LOC_Os08g14830 | NB-ARC domain containing protein | 3.54 | 6.20 | 1.75 | 1 | - | - | - | - |
| LOC_Os08g28570 | resistance protein, putative, expressed | 3.43 | 5.60 | 1.63 | 1 | - | - | - | - |
| LOC_Os08g31780 | MLA1, putative, expressed | 2.17 | 0 | 0 | 1 | - | - | - | - |
| LOC_Os10g06740 | receptor-like protein kinase precursor, putative, expressed | 5.20 | 7.51 | 1.44 | 1 | - | - | - | - |
| LOC_Os10g07566 | LRR19, putative | 0.90 | 4.07 | 4.53 | 0.92 | - | - | - | - |
| LOC_Os10g07574 | transposon protein, putative, unclassified | 4.77 | 8.91 | 1.87 | 1 | - | - | - | - |
| LOC_Os10g32990 | receptor-like protein kinase 2 precursor, putative, expressed | 17.50 | 16.71 | 0.96 | 1 | - | - | - | - |
| LOC_Os11g37759 | stripe rust resistance protein Yr10, putative, expressed | 984.96 | 795.40 | 0.81 | 0.99 | - | - | - | - |
| LOC_Os11g37850 | stripe rust resistance protein Yr10, putative, expressed | 31.98 | 23.45 | 0.73 | 1 | - | - | - | - |
| LOC_Os11g37870 | stripe rust resistance protein Yr10, putative, expressed | 14.85 | 9.27 | 0.62 | 0.99 | - | - | - | - |
| LOC_Os11g37880 | stripe rust resistance protein Yr10, putative, expressed | 1.75 | 4.45 | 2.54 | 1 | - | - | - | - |
| LOC_Os11g45620 | rust-resistance protein Lr21, putative | 31.7 | 26.79 | 0.85 | 1 | - | - | - | - |
| LOC_Os11g45970 | NBS-LRR disease resistance protein, putative, expressed | 8.63 | 12.62 | 1.46 | 1 | - | - | - | - |
| LOC_Os11g45980 | NBS-LRR type disease resistance protein, putative, expressed | 5.12 | 4.68 | 0.91 | 1 | - | - | - | - |
| LOC_Os12g09240 | NBS-LRR disease resistance protein, putative | 1.78 | 2.53 | 1.42 | 1 | - | - | - | - |
| LOC_Os12g29690 | NBS-LRR disease resistance protein, putative | 7.80 | 6.43 | 0.82 | 1 | - | - | - | - |
| LOC_Os12g29710 | NBS-LRR disease resistance protein, putative | 9.52 | 6.17 | 0.65 | 1 | - | - | - | - |
| LOC_Os12g31620 | disease resistance protein RPM1, putative, expressed | 3.86 | 7.07 | 1.83 | 1 | - | - | - | - |
| **RGAs with expression ratio of at least two among infected GV and infected VN** | | | | | | | | | |
| LOC_Os02g18510 | stripe rust resistance protein Yr10, putative | 10.27 | 35.07 | 3.41 | 0.09 | 6.27 | 0 | 0 | 0.17 |
| LOC_Os10g04674 | disease resistance protein RPM1, putative, expressed | 143.42 | 141.34 | 0.99 | 1 | 212.12 | 0 | 0 | 0.00 |
| LOC_Os11g10550 | NBS-LRR disease resistance protein, putative, expressed | 27.07 | 83.07 | 3.07 | 0.05 | 48.20 | 0 | 0 | 0.03 |
| LOC_Os11g12050 | NBS-LRR type disease resistance protein, putative, expressed | 853.24 | 927.33 | 1.09 | 1 | 89.07 | 0 | 0 | 0.02 |
| LOC_Os11g36410 | NBS-LRR type disease resistance protein, putative, expressed | 14.39 | 12.19 | 0.85 | 1 | 25.34 | 0 | 0 | 0.04 |
| LOC_Os11g39190 | NB-ARC domain containing protein, putative, expressed | 290.11 | 99.91 | 0.34 | 0.51 | 713.08 | 0 | 0 | 0.00 |
| LOC_Os11g45790 | NB-ARC domain containing protein, expressed | 354.66 | 552.15 | 1.56 | 0.58 | 242.87 | 0 | 0 | 0.00 |
| LOC_Os12g37770 | RGH1A, putative, expressed | 35.66 | 74.85 | 2.10 | 0.26 | 51.8 | 0.43 | 0.01 | 0.04 |
| LOC_Os11g39320 | LZ-NBS-LRR class, putative, expressed | 310.34 | 210.10 | 0.68 | 0.66 | 517.36 | 2.02 | 0.00 | 0.01 |
| LOC_Os11g39310 | NB-ARC domain containing protein, expressed | 331.09 | 173.24 | 0.52 | 0.12 | 702.60 | 1.81 | 0.00 | 0.01 |
| LOC_Os10g17690 | NB-ARC domain containing protein, expressed | 3.01 | 7.05 | 2.34 | 0.98 | 5.72 | 0.27 | 0.05 | 0.39 |
| LOC_Os11g37740 | stripe rust resistance protein Yr10, putative, expressed | 169.08 | 181.56 | 1.07 | 1 | 4.52 | 9.07 | 2.01 | 0.92 |
| LOC_Os11g12240 | retrotransposon protein, putative, unclassified, expressed  (Similar to NBS-LRR disease resistance protein homologue [Oryza sativa Japonica Group]) | 44.53 | 86.12 | 1.93 | 0.33 | 0.85 | 5.62 | 6.64 | 0.54 |
| LOC_Os11g37774 | stripe rust resistance protein Yr10, putative, expressed | 14.39 | 10.7 | 0.74 | 1 | 4.75 | 0.70 | 0.15 | 0.61 |
| LOC_Os11g45130 | pollen signalling protein with adenylyl cyclase activity, putative, expressed  (putative disease resistance protein RGA3-like [Brachypodium distachyon]) | 84.26 | 85.14 | 1.01 | 1 | 20.21 | 5.85 | 0.29 | 0.31 |
| LOC_Os09g19280 | retrotransposon protein, putative, unclassified  (disease resistance protein RPP13-like [Brachypodium distachyon]) | 35.41 | 55.54 | 1.57 | 0.74 | 1.41 | 5.31 | 3.77 | 0.74 |
| LOC_Os07g04900 | NBS-LRR disease resistance protein, putative | 1.39 | 6.70 | 4.83 | 0.61 | 11.99 | 0.70 | 0.06 | 0.14 |
| LOC_Os01g57340 | rp1, putative, expressed | 98.48 | 124.40 | 1.26 | 1 | 12.72 | 14.41 | 1.13 | 1 |
| LOC_Os11g11990 | NB-ARC domain containing protein, expressed | 231.17 | 423.31 | 1.83 | 0.57 | 182.08 | 59.36 | 0.33 | 0.45 |
| LOC_Os04g43440 | NB-ARC/LRR disease resistance protein, putative, expressed | 344.95 | 809.94 | 2.35 | 0.66 | 67.91 | 115.14 | 1.70 | 0.37 |
| LOC_Os11g37860 | stripe rust resistance protein Yr10, putative, expressed | 119.76 | 258.87 | 2.16 | 0.18 | 5.66 | 38.96 | 6.88 | 0.01 |
| LOC_Os08g05440 | NB-ARC domain containing protein | 130.06 | 236.32 | 1.82 | 0.2 | 67.00 | 38.63 | 0.58 | 0.39 |
| LOC_Os11g12040 | disease resistance protein RPM1, putative, expressed | 35.22 | 57.96 | 1.65 | 0.83 | 4.63 | 9.84 | 2.13 | 0.79 |
| LOC_Os01g72680 | disease resistance protein RPS2, putative | 0.80 | 22.85 | 28.42 | 0.13 | 1.26 | 3.96 | 3.15 | 0.84 |
| LOC_Os11g46210 | NB-ARC domain containing protein, expressed | 261.76 | 305.70 | 1.17 | 1 | 176.49 | 56.53 | 0.32 | 0.15 |
| LOC_Os11g45930 | NBS-LRR type disease resistance protein, putative, expressed | 175.03 | 298.38 | 1.71 | 0.38 | 44.24 | 62.62 | 1.42 | 0.72 |
| LOC_Os12g17410 | NB-ARC domain containing protein | 48.41 | 27.65 | 0.57 | 0.63 | 6.44 | 6.21 | 0.96 | 1 |
| LOC_Os11g39330 | NB-ARC domain containing protein, putative | 31.58 | 14.57 | 0.46 | 0.43 | 23.25 | 3.90 | 0.17 | 0.29 |
| LOC_Os08g10430 | NBS-LRR disease resistance protein, putative, expressed | 132.42 | 205.97 | 1.56 | 0.64 | 40.66 | 60.40 | 1.49 | 0.77 |
| LOC_Os11g39160 | NBS-LRR disease resistance protein, putative | 38.13 | 19.16 | 0.50 | 0.68 | 54.59 | 5.70 | 0.10 | 0.17 |
| LOC_Os01g70080 | NB-ARC domain containing protein, expressed | 375.95 | 1082.63 | 2.88 | 0.34 | 118.98 | 357.42 | 3.00 | 0.11 |
| LOC_Os03g20840 | disease resistance RPP13-like protein 1, putative, expressed | 78.16 | 331.62 | 4.24 | 0.09 | 99.63 | 130.61 | 1.31 | 0.76 |
| LOC_Os11g11790 | NBS-LRR type disease resistance protein, putative, expressed | 660.94 | 741.07 | 1.12 | 1 | 379.78 | 309.28 | 0.81 | 0.72 |
| LOC_Os01g06870 | resistance protein SlVe1 precursor, putative, expressed | 206.43 | 335.10 | 1.62 | 0.92 | 113.61 | 142.84 | 1.26 | 0.97 |
| LOC_Os11g03650 | mla1, putative, expressed | 1.53 | 4.14 | 2.71 | 1 | 3.78 | 1.91 | 0.51 | 0.97 |
