# Supplementary material for: Comparative Transcriptome Profiling of the Early Response to Magnaporthe oryzae in Durable Resistant vs Susceptible Rice (Oryza sativa L.) Genotypes
Source: PLoS One. 2012 Dec 12;7(12):e51609. doi: 10.1371/journal.pone.0051609 (PMC3520944; doi:10.1371/journal.pone.0051609)
Supplement: Table S11 — List of the gene loci whose transcription profile was evaluated by qRT-PCR. For each gene the forward (fw) and reverse (rev) primer sequences are provided. (DOC) [file pone.0051609.s013.doc]

**Table S11.** List of the gene loci whose transcription profile was evaluated by qRT-PCR. For each gene the forward (fw) and reverse (rev) primer sequences are provided

| **LOCUS** | **5’-3’ primer sequences** |
| --- | --- |
| LOC_Os11g02530-fw | CTGGCATCATGTCAAGAAGCTT |
| LOC_Os11g02530-rev | CCGTCACATGTTCACCATCAG |
| LOC_Os11g02540-fw | TCGCCGCCGGTGATT |
| LOC_Os11g02540-rev | ACACCACCGTCCGTACTACCA |
| LOC_Os03g08410-fw | TCGAGTCGTACCTGTCATGGAA |
| LOC_Os03g08410-rev | GGGTGATCCGGCCTCAAC |
| LOC_Os01g71474-fw | CGCCGGCCTCCTTGA |
| LOC_Os01g71474-rev | AAGAAGACGGAGACGTACGTGTT |
| LOC_Os02g56420-fw | CGCCGCTACTCGTAACATGA |
| LOC_Os02g56420-rev | TCGATGCAGTGGCTGTTGAC |
| LOC_Os05g33130-fw | AACCAGAGGCCGTTCAACAG |
| LOC_Os05g33130-rev | TTTCTCGTCGTCAGTCGTCACT |
| E2 ubiquitin-conjugating enzyme-fw (Acc. n. AK059694) | CCGTTTGTAGAGCCATAATTGCA |
| E2 ubiquitin-conjugating enzyme-rev (Acc. n. AK059694) | AGGTTGCCTGAGTCACAGTTAAGTG |
